# Exploring the Impact of Nutrition on Non-Alcoholic Fatty Liver Disease Management: Unveiling the Roles of Various Foods, Food Components, and Compounds

**DOI:** 10.3390/nu15132838

**Published:** 2023-06-22

**Authors:** Marcin Kosmalski, Rafał Frankowski, Kacper Deska, Monika Różycka-Kosmalska, Tadeusz Pietras

**Affiliations:** 1 Department of Clinical Pharmacology, Medical University of Lodz, 90-153 Lodz, Poland; tadeusz.pietras@umed.lodz.pl; 2 Students’ Research Club, Department of Clinical Pharmacology, Medical University of Lodz, 90-153 Lodz, Poland; rafal.frankowski@stud.umed.lodz.pl (R.F.); kacper.deska@stud.umed.lodz.pl (K.D.); 3 Department of Electrocardiology, Medical University of Lodz, 92-213 Lodz, Poland; monika-rozycka-kosmalska@umed.lodz.pl

**Keywords:** non-alcoholic fatty liver disease, nutrition, food treatment, supplementation

## Abstract

There is a need to introduce standardized treatment options for non-alcoholic fatty liver disease (NAFLD) due to its global prevalence and the complications of this disease. Many studies have revealed that food-derived substances may be beneficial in dealing with this disease. Therefore, this review aims to evaluate the recently published studies on the food-derived treatment options for NAFLD. A comprehensive search of the PubMed database using keywords such as “NAFLD”, “nutrition”, “food”, “derived”, “therapy”, and “guidelines” yielded 219 relevant papers for our analysis, published from 2004 to 2023. The results show the significant benefits of food-derived treatment in NAFLD therapy, including improvements in liver histology, hepatic fat amounts, anthropometric measures, lipid profile, and other metabolic measures. The availability of the substances discussed makes them a significant adjuvant in the treatment of this disease. The usefulness of Viusid as additional therapy to diet and physical activity should be emphasized due to improvements in liver histology; however, many other substances lead to a decrease in liver fat amounts including, e.g., berberine or omega-3 fatty acids. In addition, the synbiotic Protexin seems to be useful in terms of NAFLD treatment, especially because it is effective in both obese and lean subjects. Based on the latest research results, we suggest revising the therapeutic recommendations for patients suffering from NAFLD.

## 1. Introduction

NAFLD represents a serious global problem due to the complications it brings and its rising prevalence worldwide. The prevalence of NAFLD is estimated at about 38% in the global population and it is higher in males than females (circa 40% vs. circa 26%). NALFD is considered the most frequent liver disease and the main factor of mortality and morbidity related to liver diseases [1].

**Figure 1:**
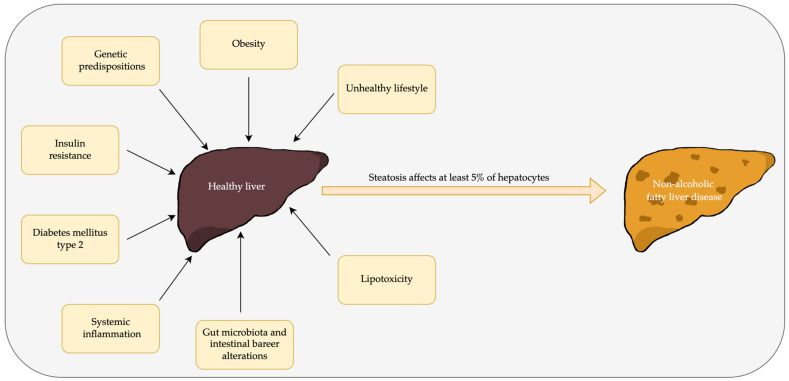
A brief overview of the NAFLD pathophysiology.

The pathogenesis of NAFLD is complex. The factors leading to its development include obesity, which is also associated with insulin resistance (IR) and diabetes, lifestyle, and inflammatory process, including oxidative stress. All of these factors are indisputably related to each other [2,3,4]. The causes of the development of NAFLD in obese patients can be seen, for example, in the inflammatory process. Fatty tissue is responsible for the secretion of proinflammatory cytokines such as interleukin (IL) 6, IL-8, or tumor necrosis factor alpha (TNF-α), which affect liver condition, exacerbate NAFLD, and promote its progression to non-alcoholic steatohepatitis (NASH). It was also observed that in obese subjects, anti-inflammatory pathways are decreased. Inflammation disturbs the Kupffer cells’ function in the liver and leads to an exacerbation of the inflammatory process in this organ and promotes oxidative stress. In addition, as a consequence of these processes, hepatic stellate cells become activated, resulting in liver fibrosis [5]. Visceral adipose tissue is known as a promotor of lipotoxicity for which its role in liver destruction has been proven [6]. The evidence of the gut microbiota’s role in the pathogenesis of NAFLD indicates that it may be related to systemic inflammation generated by alterations in intestinal permeability. This is a result of the passage of metabolites and pro-inflammatory cytokines through the injured intestinal barrier [7,8,9]. Genetic factors are also important in the pathogenesis of NAFLD, among which genes responsible for lipid metabolism can be distinguished, for example, *PNPLA3* (patatin-like phospholipase domain-containing 3) and its polymorphisms, or *TM6SF2* (transmembrane 6 superfamily member 2) [10]. It was also observed that fructose intake has an impact on the expression of genes related to the pathogenesis of metabolic disorders, e.g., *Fas* (fatty acid synthase) or *PPARα* (peroxisome proliferator-activated receptor α) [11]. Nevertheless, patients who are not overweight also suffer from this disease, which shows how complex NAFLD development is [12]. As obesity is one of the major factors in NAFLD development, it is not surprising that weight loss leads the way in dealing with this condition. A lot of studies have proven its effectiveness, with or without additional strategies such as diet or medications, e.g., antidiabetic drugs. What is related to optimal body weight maintenance is physical activity. There is strong evidence that this intervention is effective in NAFLD treatment. A lot of studies investigate NAFLD treatment through physical activity combined with dietary intervention. It is worth emphasizing that it is effective in reducing liver fat amounts even when weight loss is not observed. A beneficial impact on the NAFLD state achieved by physical activity is seen through improvements in the intestinal microbiota, weight loss, and impacts on epigenetic factors [13]. A short graphic presentation of the NAFLD pathogenesis mechanism and the risk factors leading to it is presented in Figure 1.

Evidence of at least 5% fatty hepatocytes without any identifiable secondary cause of this condition constitutes the diagnosis of this disease. Among the complications of this state is a progression to nonalcoholic steatohepatitis, in which liver fibrosis and hepatocyte ballooning are observed, as are cirrhosis, kidney, and cardiovascular diseases. In addition, an increased risk of malignant tumors, of which the most frequent is hepatocellular carcinoma, due to NAFLD was observed [14,15,16].

NAFLD diagnostics can be divided into invasive and non-invasive. The first one is a liver biopsy which is a gold standard in NAFLD staging. When it comes to non-invasive procedures, imaging and laboratory tests can be specified. Among imaging tools, ultrasound, transient elastography, magnetic resonance, Xeon-133 liver scan, and computed tomography are present. Laboratory tests contain tools used to calculate scales such as the NAFLD liver fat score (NLFS), SteatoTest, NAFL screening score, lipid accumulation product (LAP), hepatic steatosis index (HSI), or fatty liver index (FLI). The secondary causes of lipid accumulation in the liver should be excluded in order to make an NAFLD diagnosis. Recently, it was observed that irisin may be a diagnostic marker of NAFLD [17].

Even though the NAFLD problem is well known, we cannot find FDA-approved drugs in Europe to treat this disease; there are only recommendations for a healthy lifestyle and diet, and the medication guidelines refer mainly to NASH [18,19]. In our previous work, we proposed a path of NAFLD treatment with diet, physical activity, and pharmacotherapy [13]. Now, we aim to take a closer look at food-derived substances in NAFLD supplementary therapy.

Studies are showing the effectiveness of the use of certain drugs in NAFLD treatment, e.g., dapagliflozin or pioglitazone [20,21]. Dietary supplements are also considered a potential therapeutic point of this disease, states about the basis of this study which contains the review of the most actual literature on the effect of functional foods and dietary supplements on liver function and NAFLD. Functional foods are described as natural or industrially-processed foods that may have a beneficial outcome on health, beyond basic nutrition [22]. Diet supplements which are used to improve human health are named nutraceuticals. Nowadays, they seem to act as therapeutic options for many diseases. Nutraceuticals may play a greater role considering that many people do not receive enough micronutrients from their food [22,23].

A short specification of each substance is given in Section 3, and information about the study group and the specific interventional impacts on NAFLD is contained in the tables in Section 3.1, Section 3.2 and Section 3.3.

Our review aims to determine of the suitability of food-derived supplements as a supportive therapy for NAFLD and the collective ordering of the influence of given substances on individual parameters among NAFLD subjects.

## 2. Materials and Methods

### 2.1. Focal Question

The determination of the best food-derived supplementary therapeutic options for NAFLD by summarizing the results of different studies in the field of food-product-derived additional therapy in NAFLD was set as the primary aim of this review.

### 2.2. Language

Studies published in English were included in the analysis.

### 2.3. Databases

The PubMed database, according to Preferred Reporting Items for Systematic Reviews and Meta-Analyses (PRISMA) guidelines [24] (Supplementary Table S1), was searched; the PRISMA flow diagram indicates the number of articles included and excluded (Supplementary Figure S1).

### 2.4. Study Extraction

In relation to publication date, more than 50% of the evaluated papers were published within the last 5 years. Mainly, we searched clinical trials and randomized controlled trials. The final search was performed on 2 June 2023. In order to find matching articles, we used a combination of Medical Subject Heading (MeSH) terms and specific search terms in variations, including “non-alcoholic fatty liver disease”, “NAFLD”, “nutrition”, “food”, “derived”, “therapy”, and “guidelines”. The relevant works fulfilling the following inclusion criteria were selected: English language studies on NAFLD supplementary treatment strategies involving food-derived and nutraceutical substances. All the selected studies were then downloaded and analyzed.

### 2.5. Data Extraction

Initially, titles, abstracts, or full texts were reviewed; subsequently, full copies were analyzed. In relation to the prepared and tested template, data on the effects of used substances as NAFLD treatment options, a description of the substance used, study populations, NAFLD diagnostic methods, and general information about the disease were extracted from the articles. The results are reported in different paragraphs, containing a short specification of each substance and its results on the parameters of NAFLD-diagnosed patients. Analyses were performed using Microsoft Office 365, CiteSpace (v.6.1. R2), and VOSviewer (v.1.6.18) to conduct a bibliometric and visual analysis of all data.

### 2.6. Quality Assesment

The final assessment and verification were carried out by experienced researchers (M.K., M.R.-K. and T.P.).

## 3. A Brief Description of the Substances and Their Usefulness in NAFLD Therapy

### 3.1. Plants

#### 3.1.1. Silybin

Silybin is an ingredient acquired from milk thistle (*Silybum marianum*) silymarin extract and constitutes 50–80% of its composition. In traditional medicine, milk thistle properties have been used for over 2000 years to improve liver condition. It is known that silybin manifests a strong antioxidant effect; moreover, it has been proven that it has immunomodulatory properties and presents a beneficial effect on metabolic parameters such as preventing diabetes mellitus or decreasing the cardiovascular risk. The hepatoprotective action of milk thistle extracts was also observed [25,26].

Animal models have proven that silybin decreases the inflammatory state and fibrosis in NASH and is responsible for an improvement in glucose tolerance in diabetes mellitus type 1 (T1DM). An improvement in glucose levels among diabetes mellitus type 2 (T2DM) patients was also observed [27,28]. Loguercio et al. [29] performed a randomized controlled trial (RCT) study and revealed the beneficial impacts of realsil, which is composed of silybin and phosphatidylcholine, in conjunction with vitamin E supplementation in NAFLD patients. Improvements in the liver histopathologic image, insulin sensitivity, and normalization of hepatic parameters were observed due to this action [29]. A similar study proved that silymarin administration may improve liver fibrosis in regard to histology, and liver stiffness measurements [30]. The other study proved that silybin use resulted in a decrease in total cholesterol (TC), triglycerides (TG), ALT, AST, GGT, and fasting plasma glucose, due to a 16-week follow-up supplementation of 3 × 35 mg three times per day [31].

#### 3.1.2. Danshao Shugan Granules (DSSG)

DSSG comes from traditional Chinese medicine (TCM) and was registered officially in China as a substance with hepatoprotective activity which decreases the fat accumulation in the liver. However, the DSSG mechanism of action on hepatocytes remains unclear. In the literature, there are only a few articles about this substance. However, Wang et al.’s [31] study proved its efficiency in NAFLD. At the endpoint of this intervention, improvements in liver B-ultrasonography, lipid profile, and liver enzymes were observed. This study was also conducted using a rat model, which revealed that DSSG reduced malondialdehyde and NF-κB activity and increased the activity of superoxide dismutase. These changes may indicate the mechanisms of DSSG action. Liver improvements, measured via a B-ultrasound for DSSG treatment, were greater following DSSG treatment compared to silybin or rosiglitazone [31].

Another substance used in TCM is Danshao Huaxian (DSHX). In rat models following DSHX implementation, an improvement in liver indices was observed. ALT and hyaluronic acid levels declined, and liver fibrosis intensity was reduced. It was also revealed that the activity of metalloproteinase-1 (MMP-1) was elevated and that a reduced expression of tissue inhibitor of metalloproteinase-1 (TIMP-1) was observed due to an intervention with an 8-week DSHX follow-up. According to MMP-1 and TIMP-1, it is known that alterations in their connections play a role in liver disease pathogenesis, by, e.g., the degeneration of extracellular matrix disturbances, or the fact that TIMP-1 is elevated in cirrhosis [32]. Another route of DSHX’s mechanism of action in liver disorders involves an increase in BMP-7 expression and decrease in Gremlin expression. This explanation is given by another study in which the effectiveness of using this therapy, measured by a decrease in ALT, AST, improvement in liver histology and decrease in liver homogenate transforming growth factor β1 (TGF- β1) levels, was confirmed in a rodent model [33]. An improvement in liver function as a result of using DSHX was also obtained in a study on patients with hepatitis B virus (HBV)-related cirrhosis. The researchers discovered an increase in serum albumin, and a decrease in serum bilirubin and the Child–Pugh scale score. Furthermore, decreased HBV DNA copies in the serum were detected [34].

#### 3.1.3. Dihydromyricetin

*Ampelopsis grossedentata* has been utilized for centuries in TCM. The main ingredient of it is a dihydromyricetin (the second name is ampelopsin); the dihydromyricetin content in the leaves of this plant can reach 30%. Its anti-inflammatory and anti-oxidative properties have been used for dealing with frequent infections such as a sore throat, chills, or even viral liver diseases, which may indicate its hepatoprotective activity. Beneficial outcomes due to dihydromyricetin supplementation were also observed in relation to the lipid profile [35,36]. According to the mentioned beneficial outcomes of this substance’s use, researchers conducted a study on its supplementation in NAFLD patients with satisfactory results when it comes to lipid profile, liver enzyme levels, or glycemic parameters [37].

#### 3.1.4. Anthocyanin

Anthocyanin is a plant-derived compound that is obtained from blueberries (*Vaccinium myrtillus*) and black currants (*Ribes nigrum*) and has positive effects on liver health when used as a supplement. For this substance, antioxidant and anti-inflammatory activities were revealed in subjects with dyslipidemia. Due to anthocyanin supplementation, the levels of IL-6 and TNF-α declined, and the parameters of oxidative stress, such us malonaldehyde (MDA), were improved, indicating its beneficial outcomes on the treatment of metabolic diseases [38]. In addition, there was a reduction in body fat and improvement in superoxide dismutase (SOD) activity. The importance of this substance in improving the lipid profile is also a subject of research; however, Bakuradze et al. [39], through their study on fruit juice rich in anthocyanin, revealed that these effects may be related to the high doses of vitamin C in this juice [39]. Due to these facts, a study on its impact on the NAFLD state was conducted and the results showed its beneficial outcomes in NAFLD-diagnosed patients when it came to liver function and glucose maintenance parameters [40].

#### 3.1.5. Chia

Chia seed (*Salvia hispanica* L.) popularity has been growing recently. This popularity is more importantly reflected in the performance of these seeds, which are composed of high amounts of fiber, protein, positive fatty acids, and antioxidants. Regarding the fatty acids present in chia, α-linoleic acid (ALA) and other linoleic acids are known to be protective for metabolic disorders [41]. It is known that supplementation with omega-3 fatty acids, among which ALA is included, aids in reducing the fatty amounts in the liver in NAFLD patients, and, within subjects with T2DM, it reduces the systolic blood pressure (SBP) [41,42]. A study in which chia supplementation was included in the diets of subjects with NAFLD showed the regression of NAFLD in about 52% of participants, which may be related to lipid profile improvement, bodyweight decrease, and serum ALA increase. In addition, chia supplementation may be a good strategy for NAFLD due to its potentially preventive action against NAFLD progression or the development of metabolic complications [43].

#### 3.1.6. *Zataria Multiflora*

The plant *Zataria multiflora* (ZM), or shirazian thyme, is grown in Iran, Pakistan, and Afghanistan. It has similar pharmacological aspects to Thymus vulgaris. This herb contains phenols and flavonoids, the main ingredients of which are thymol and carvacrol. Within the substances in the ZM ingredients list, we can also specify borneol, p-cymene, linalool, caryophyllene, and γ-terpinene. Locally, it is used as a traditional medicine component because of its antiseptic, anti-inflammatory, and antioxidative actions. The hepatoprotective and antidiabetic functions of this herb have also been revealed [44,45,46]. When it comes to the anti-inflammatory action of ZM, it is a result of the reduction in proinflammatory cytokines (for example IL-4) and oxidative stress, with an increase in anti-inflammatory cytokines (for example IFN-γ) and antioxidant levels. These changes are related to gene expression fluctuations as a result of treatment [47]. In regard to the clinical aspects of ZM, a study on NAFLD was also conducted; it showed significant reductions in insulin level, insulin resistance, and blood pressure as a result of the supplementation. However, this study did not prove any significant differences when it came to liver enzymes, fatty liver measures in USG, or lipid profiles in comparison to the placebo group [48]. These data are partially in line with another study, in which a *Zataria*–oxymel mixture was administered. In obese patients, there were no differences in lipid profile and liver enzymes due to the supplementation. However, reductions in waist circumference, hip circumference, and HOMA-IR were observed [49]. In sum, further studies are needed to explore ZM’s influence on NAFLD.

#### 3.1.7. Fenugreek

Fenugreek (*Trigonella foenum-graecum* Linn.) is widely used as a spice in many countries. As it has beneficial outcomes on health, it is used in traditional medicine, including TCM. These favorable effects have been proven by scientific research, and include antidiabetic, anti-hypercholesterolemic, anti-inflammatory, antioxidant, antiseptic, and hepatoprotective actions. Regarding the composition of fenugreek, alkaloids, polyphenols, flavonoids, lipids, amino acids, and steroids can be specified [50,51]. A study conducted by Geberemeskel et al. [52] on 114 newly diagnosed T2DM patients in which fenugreek seed powder was used for treatment showed beneficial outcomes, even in comparison to metformin (control group). This improvement in lipid profile in comparison to baseline and compared with the control group was statistically significant, while changes in lipid profile in metformin follow-up patients in relation to baseline did not reach significance. Furthermore, an increase in HDL was significantly observed only in fenugreek-treated subjects [52]. One meta-analysis indicated fenugreek’s antidiabetic and TC-lowering effects; however, it also revealed that changes in TG, LDL, and HDL did not reach significance and further studies are needed to prove or disprove fenugreek’s efficiency in relation to this matter [50]. A study on NAFLD patients who were given fenugreek seed hydroalcoholic extract, at a dose of 1 g per day for 3 months, was also conducted; however, its results did not clearly indicate the beneficial outcomes due to this strategy. Importantly, in the intervention group, only 13 subjects were analyzed, and all of them were males. However, it is important to say that even if the results were not statistically significant, the reduction in liver steatosis in the treatment group was higher compared to placebo [53].

Due to the lack of studies on numerous subjects with NAFLD, we cannot decide if this strategy of treatment is efficient in this disease; however, based on studies in other patient groups, positive effects of such interventions might be expected. Thus, this can provide a basis for further studies.

#### 3.1.8. *Nigella sativa*

The next plant which is commonly used as a treatment seedling is *Nigella sativa* (NS), which is also known as black cumin or black seed. The most frequent places of its occurrence are in Europe and Asia. NS is often used traditionally in the Middle East and Southeast Asia, including in the treatment of asthma, hypertension, diabetes, cough, bronchitis, and gastrointestinal diseases [54]. Additionally, the beneficial outcomes related to liver steatosis, the lipid profile, and the antidiabetic, antioxidant, and anti-inflammatory activities of NS are known. The positive effects of NS supplementation on NAFLD are supported by a meta-analysis, which revealed an improvement in NAFLD harshness, liver enzyme levels, hs-CRP, fasting plasma glucose (FPG), and HDL. Even if these results were significant, the authors indicate that due to heterogeneity of them, these outcomes should be interpreted with caution [55]. Among the overweight females, a reduction in cardiovascular disease by ameliorating its risk factors, e.g., lipid profile improvement or systolic blood pressure decrease, was observed due to NS supplementation [56]. A significant reduction in the liver steatosis grade, improvement in BMI, body weight, liver enzymes, lipid profile, and a decrease in inflammatory biomarkers were observed due to NS supplementation in a few studies conducted on NAFLD patients, and the authors of these studies indicate its preventative role in NAFLD progression [57,58,59].

On the other hand, Rashidmayvan et al. [54], in a study on 44 NAFLD patients, did not reveal significant differences in cardiovascular risk factor parameters between placebo and NS oil supplementation in a dose of 1 g per day follow-up for 8 weeks. The measured parameters were leptin, adiponectin, and blood pressure. The comparison within groups also did not reveal a significance in parameter changes [54]. In sum, there is a need to conduct more studies in this field.

#### 3.1.9. *Camelina sativa*

*Camelina sativa* is an oilseed plant cultivated in Europe, Asia, and Northern America for organic oil production, as a medical form, and as a raw material used in the energy industry. This shrub has been known for thousands of years and was cultivated already in the times Before the Common Era. It is also known as a linseed dodder, gold-of pleasure, or German sesame [60]. It was revealed that *Camelina sativa* oil (CSO) enrichment in the diet improves the lipid profile and has immunomodulatory effects [61,62,63] In the CSO, ALA makes up about 38% of the composition for which beneficial health outcomes are known [56]. Due to the properties of this plant, it was tried to use it in NAFLD therapy with satisfactory results, including improvements in WC, BMI, TG, TC, LDL, and ALT. The authors indicate its possible anti-inflammatory and beneficial effects on oxidative stress parameters, and efficiency in NAFLD patients [64]. Other researchers have linked CSO supplementation with prebiotics. Significant HOMA-IR, high specific C-reactive protein (hs-CRP), cortisol, and malondialdehyde (MDA) decreases were observed, and total antioxidant capacity (TAC) and superoxide dismutase (SOD) levels were increased [65].

#### 3.1.10. *Garcinia cambogia*

Garcinia species are native to tropical regions such as Asia, Brazil, and Africa. Due to the presence of xanthones, bioflavonoids, polyphenols, and benzophenones in the Clusiaceae species family, which includes the Garcinia species, the effects of this plant on a variety of diseases are currently being investigated. Studies have revealed its antiseptic, antioxidant, and anti-inflammatory effects on organisms. Even action against tumor progression was observed [66]. The main active ingredient of this plant is a hydroxycitric acid (HCA) which can block ATP-citrate lyase and decrease lipogenesis. A study on obese subjects revealed that supplementation with garcinia extract at a dose of 2.4 g per day for 60 days decreased TG levels, which may be linked to lipogenesis inhibition [67]. Another study, in obese subjects, in which a combination of *Garcinia cambogia* with other substances—*Camellia sinensis*, unroasted *Coffea arabica*, and *Lagerstroemia speciosa*—named IQP-GC-101 was taken in conjunction with a calorie-restricted diet revealed that weight loss, body fat mass, and waist and hip circumference reduction were side effects of this treatment. These results were shown after 14 weeks of the intervention, and were compared to placebo combined with the same diet [68]. The effect of HCA supplementation on NAFLD was also investigated. In NAFLD patients, there was an improvement in anthropometric measures, glucose levels, and the lipid profile [69]. Even if a positive impact of Garcinia was shown, there were a lack of studies investigating its impact on liver steatosis in NAFLD.

#### 3.1.11. Chlorella Vulgaris

Microalgae *Chlorella vulgaris* has been used for centuries, especially in Asia, as a functional food and for therapy due to its antibacterial and anti-inflammatory actions. It is a source of vitamins, amino acids, fatty acids, minerals, fiber, and a lot of phytochemical compounds. It is considered that Chlorella vulgaris may decrease the cardiovascular risk, and have a beneficial impact, e.g., on diabetes, atherosclerosis, and anemia. In rodent studies, a reduction in ALT and AST levels was found, which may be linked to the antioxidant activity of this algae [70,71]. Studies examining patients with NAFLD revealed that its supplementation, combined with metformin or vitamin E use, results in patient state improvement. In the Panahi et al. [72] study, reductions in BMI, weight, liver enzymes, TG, glycated hemoglobin (HbA1c), HOMA-IR, and uric acid levels were seen. These results are in line with Ebrahimi-Mameghani et al.’s [71] study, which also revealed improvements in weight, liver enzymes, and, in addition, a decrease in alkaline phosphatase (ALP). However, in this study, the decrease in FPG and improvement in lipid profile were significant, while in the Panahi et al. study, the FPG changes were borderline significant, and improvements in LDL and HDL were not observed [71,72].

#### 3.1.12. Coffee

Coffee is a brew derived from roasting coffee beans harvested from coffee trees. The major cultivated species are *Coffea arabica* L. and *Coffea canephora*. These plants are cultivated in tropical areas. The popularity of coffee worldwide is not a surprising fact due to its taste and stimulating qualities as a result of having a high concentration of caffeine. The coffee tree is cultivated in many tropical countries. However, these beans have more important effects on human life including decreasing the risk of diabetes development and neurological complications. The risk of cancer may be reduced in subjects consuming coffee, but this needs further investigation. Within the components of coffee, caffeine, chlorogenic acids, polyphenols, alkaloids, vitamins, metals, and terpenoids can be specified [73,74]. In the literature, studies revealing the effects of coffee use in NAFLD patients can be found. As caffeine and chlorogenic acids make up a significant part of coffee components, researchers have investigated their influence on patients with NAFLD and diabetes. The intervention was set as supplementation with caffeine and chlorogenic acids in doses of 200 mg per day, respectively, to groups. At the endpoint, a reduction in TC was observed in the caffeine-supplemented group, and an increase in insulin levels was associated with co-supplementation with caffeine and chlorogenic acid. However, the alleviation of liver fat amounts, or fibrosis, was not observed [75]. However, Cossiga et al.’s [76] study on NAFLD patients found that supplementing with a combination of *Berberis aristata*, *Elaeis guineensis*, and decaffeinated green coffee using *Coffea canephora* extracts improves glycemic control, and, more importantly, liver steatosis, as measured by elastography with a controlled attenuation parameter (CAP) [76]. Beneficial outcomes were also observed at follow-up due to green coffee extract supplementation. At the endpoint of this intervention, a reduction in BMI, TC, and TG, and increase in HDL were shown. However, these changes may be related to BMI decline, and, after adjustment, the TG level changes did not show significance [77].

#### 3.1.13. Green Tea

Tea is the most popular drink worldwide and has been known about in China for at least 5000 years. Tea is a drink obtained by a decoction of *Camellia sinensis* herb leaves. Its greatest components are catechins and polyphenols (mainly flavonols). Green tea has an antioxidative and anti-inflammatory action, and it is considered a antidiabetic and cardioprotective agent. Its anticancer activity has also been underlined [78,79,80]. The effect of green tea on NAFLD has also been studied. Sakata et al. [81] conducted a study on subjects with NAFLD, giving them green tea with low-density or high-density catechins for 12 weeks. At the endpoint, the most beneficial outcomes were observed in the high-density catechin group, including an improvement in the liver-to-spleen CT attenuation ratio, which contributes to liver state, body fat, and ALT levels. A reduction in oxidative stress, measured using urine 8-isoprostane excretion reduction, was also observed [81].

#### 3.1.14. Genistein

Genistein is a phytoestrogen and isoflavone which has its roots in TCM. Its beneficial role in decreasing breast, prostate, and lung cancer risk has been proposed. Large amounts of this substance can be found in soy, but it is known to be present also in plants such as clover sprouts, broccoli, sunflower, and cauliflower [82]. An improvement in intestinal microbiota, insulin resistance, and an increase in skeletal muscle fatty acid oxidation were observed due to genistein supplementation in obese patients [83]. Additionally, in metabolic-syndrome-diagnosed females, improvements in metabolic compounds were observed following one-year genistein supplementation. More specifically, researchers observed improvements in fasting glucose, fasting insulin, the HOMA-IR factor, and lipid profile. Even a decrease in blood pressure was associated with this intervention [84]. A rat model study showed favorable effects on glucose management, as well as genistein’s anti-inflammatory and antioxidant capabilities [85]. According to the role of genistein in organisms, researchers took a closer look at its action in NAFLD. The participants were ordered to supplement with genistein, and recommendations of physical activity and an energy-balanced diet were also given. Decreases in waist–hip ratio and insulin resistance were observed. In addition, improvements in inflammatory and oxidative stress parameters were related to genistein supplementation [86].

#### 3.1.15. Sesame Oil

Sesame oil is a product derived from *Sesame indicum* and contains monounsaturated fatty acids (MUFAs) and polyunsaturated fatty acids (PUFAs), sesamin and sesamolin. Components of sesame oil contribute to its antioxidant and anti-inflammatory activities. A mouse model revealed that sesame oil supplementation results in a reduction in atherosclerosis and leads to improvements in the lipid profile [87] and even protective action against fibrotic collagen in the liver [88]. Lipid profile enhancement, antioxidant activity, and protective action regarding blood pressure were also revealed for sesame oil in the human subject study [89]. The supplementation of sesame oil with a low-calorie diet was also tested in regard to NAFLD. The main point of this study was to determine what benefits sesame oil supplementation brings when added to the diet. Reductions in fatty liver grade and liver enzymes were observed. Improvements in anthropometric measures were also observed, but they were significant for the sunflower oil follow-up group as well. These results are based on 12 weeks of supplementation using 30 g of sesame oil with the mentioned diet in the female group. The authors indicated that sesame oil strengthens the effects of the diet and that it may be a good additional therapy for weight loss [90].

#### 3.1.16. Olive Oil

Olive oil is obtained by pressing *Olea europaea* L. fruits. Olives contain fatty acids, including MUFAs, phenolics, tocopherols, and phytosterols. Olea europaea is a tree that can be found in the Mediterranean. Additionally, olive oil makes up the basis of the Mediterranean diet. The beneficial effects of high amounts of olive oil in the diet on health are known, and can decrease the cardiovascular risk. The anti-inflammatory and antioxidative properties of this oil were also investigated [91,92]. A rodent model study on rats with NAFLD revealed that MUFAs supplementation results in a decrease in hepatic fat amounts by reducing the TG levels in this organ [93]. In the literature, a lot of randomized controlled trials on olive oil’s influence on NAFLD can be found. They generally demonstrate the positive effects of such an intervention. The positive aspects of this intervention include a reduction in BMI and the plasma levels of liver enzymes, and improvements in insulin resistance and lipid profile. Some of them highlight a reduction in fatty liver grading. Rezaei et al.’s [94] study proved that when it comes to changes in the ultrasound-measured stage of fatty liver, olive oil is more efficient than sunflower oil when combined with a hypocaloric diet. In sum, the recommendation of olive oil supplementation as an addition to a hypocaloric diet may increase the benefits of weight loss [94,95,96].

#### 3.1.17. Oat

Oat is a grain is a rich in bioactive components, which is cultivated in many countries. A lot of studies have proven its positive impact on human health. The benefits of oat in the diet are related to improvements in cardiovascular, metabolic, and gastrointestinal diseases and cancers [97,98,99,100]. A study examining NAFLD subjects has proven the positive effects of oats in regard to patient condition. Researchers compared the hypocaloric diet with oat content to the same hypocaloric diet without enrichment. At the endpoint, a higher decrease in the hepatorenal index, and in liver fat amounts, was observed in the group with oat fiber intake. A decrease in blood pressure was seen only in the oat-supplemented group [101].

#### 3.1.18. Pinitol

The components of pinitol include soy, carob, pine wood, alfalfa, and legumes. The term pinitol refers to another name for 3-O-methyl-chiro-inositol. This substance was used in the traditional medicine of the Himalayan region where the *Abies pindrow* tree exists, and also in Asia due to the existence of *Bougainvillea spectabilis.* Pinitol was discovered to have anti-inflammatory qualities as well as a good impact on glucose management and liver health [102,103,104]. A rodent model using T2DM-induced rats revealed improvements in their glucose levels and lipid profile, which confirmed the antihyperlipidemic effects of this substance [104]. This study conducted on NAFLD-confirmed humans revealed a decrease in liver lipid amounts and an improvement in oxidative stress parameters due to follow-up pinitol consumption. The results were satisfactory, but what favors a higher dose of pinitol (500 mg) over a low dose (300 mg) is the additional lowering of ALT and GGT [105].

#### 3.1.19. Sumac

Sumac is a prominent fruit of *Rhus coriaria* L. that is used as a spice. It is found and used primarily in the Mediterranean region, the Middle East, and the United States. The Rhus genus contains over 250 species. The antimicrobial, anti-inflammatory, and antioxidative properties of this fruit are known. Additionally, the hepatoprotective effects of sumac have also been considered [106]. The effects of sumac powder supplementation in patients with NAFLD were investigated and showed a reduction in liver fibrosis, measured using Fibroscan, improvements in inflammatory markers, and glucose homeostasis parameters [107].

#### 3.1.20. Berberine

Berberine is an alkaloid derived from the *Coptis chinensis French* herb and is used in TCM as an antidiabetic medication. It was revealed that its use may have LDL-lowering effects [108,109]. Within T2DM subjects, it was revealed that berberine use has a similar glucose-level-lowering effect as metformin. Not only was glycemia homeostasis improved in the berberine group, but researchers also observed a reduction in TG, TC, and LDL levels after 3 months of follow-up to supplementation [109]. It is not surprising that a study on the berberine impact of the NAFLD state was conducted. Berberine supplementation, in conjunction with lifestyle interventions, resulted in improvements in body weight, lipid profile, insulin resistance parameters, and reduced liver fat content. What is noteworthy is that in comparison to pioglitazone, in this study, berberine was more efficient in regard to weight reduction and lipid profile change aspects [110].

#### 3.1.21. Mastiha

Mastiha is a natural product containing phytosterol, terpenes, proteins, poly-β-myrcene, and phenolic substances. Mastiha is produced from the exudate of *Pistacia lentiscus* L. species, which can be found in the Mediterranean area. Its health-promoting properties have been known since ancient times in Greece. It was used to treat gastrointestinal diseases. Its anti-inflammatory and antioxidative effects have been discovered [111,112]. Mastiha’s anti-inflammatory actions are related to the preventive effects of an increase in micro-RNA 155 (miR-155) levels. MiR-155 is related to Th17 cell functioning and what follows these inflammatory processes [113]. A study conducted on NAFLD subjects revealed that mastiha supplementation positively affects intestinal dysbiosis and lipid metabolite levels. Moreover, in severely obese subjects, liver inflammation (based on iron-corrected T1 levels) and fibrosis parameters were decreased due to this intervention [114].

The studies on plant supplementation that have been conducted on NAFLD patients are presented in Table 1.

### 3.2. Fruits, Vegetables, Flowers, and Spices

#### 3.2.1. Fruits

##### Cranberry

American cranberry (*Vaccinium macrocarpon*) is a shrub cultivated mostly in North America but also in the United Kingdom, Netherlands, and Poland. It can also be found in wild areas such as swamp cranberry (*Vaccinium oxycoccus*). Cranberry fruits are rich in polyphenols, vitamins, and minerals and contain lutein and beta-carotene. Its anti-inflammatory and antioxidant properties, and beneficial impacts on the lipid profile and glycemia-related parameters are known [115,116,117,118]. When it comes to cranberry juice supplementation in terms of plasma glucose levels, the study’s results are inconsistent; some researchers observed a decrease, while others did not. However, what is associated with cranberry juice is that it improves oxidative stress parameters [115,119]. One study in NAFLD patients also linked a reduction in the degree of fatty liver to the effects of cranberry supplementation. In addition, this study showed a positive effect on the lipid profile and insulin resistance in this group of patients [120]. Hormoznejad et al.’s [121] study on NAFLD patients revealed the beneficial outcomes of cranberry supplementation combined with a weight-loss diet. A greater decrease in ALT and insulin levels was observed in the cranberry-supplemented group in comparison to the placebo group, in combination with the mentioned diet. In addition, improvements in insulin resistance were observed due to supplementation. On the other hand, it was revealed that in regard to liver fat amounts and anthropometric parameter improvements, cranberry supplementation in the diet was no more effective than diet plus placebo [121].

##### Pomegranate

Pomegranate (*Punica granatum* L.) is a plant from the Middle East; now, it is cultivated in plenty of countries around the world. Among the compounds in pomegranate, phenols, flavonoids, catechins, alkaloids, and sterols can be specified [122]. Pomegranate has been used in traditional medicine due to its anti-inflammatory, antimicrobial, and antioxidant properties. Additionally, its positive effects on the cardiovascular system and glucose maintenance have observed. A study conducted on T2DM patients revealed that pomegranate juice consumption improves the pancreatic beta-cell state, insulin resistance, and the level of glucose in the blood [123]. Additionally, its beneficial response in NAFLD patients was shown. Pomegranate extract resulted in improvements in lipid profiles, insulin resistance parameters, and even anthropometric measures [124].

##### Naringenin

Naringenin is a flavanone derived from citrus fruits such as orange and pomegranate. Its anti-inflammatory, antioxidant, weight-lowering, and lipid parameter improvement properties are known. Even anti-microbial and anti-tumor effects have been observed in certain studies on naringenin [125]. A randomized controlled trial (RCT) performed on overweight patients with NAFLD revealed that naringenin supplementation is effective when it comes to improving the lipid profile and atherogenic index of plasma. Additionally, reductions in visceral adipose tissue and SBP were shown [126]. Naringenin’s effects, when it comes to lipid profile beneficial outcomes, were proven by another study on NAFLD subjects; what is more, this RCT revealed a decrease in the NAFLD grade. However, the reduction in liver enzymes in the blood did not reach significance [127].

##### Bergamot Citrus

Bergamot (*Citrus bergamia*) is a citrus found in southern Italy, where it has been used to treat common infectious diseases such as sore throats or fevers. It is rich in flavonoids and is known for its antioxidant and anti-inflammatory properties [128]. Furthermore, it has been discovered to have a positive effect on the lipid profile [129]. Capomolla et al.’s [130] study revealed that pectin-enriched formulations of bergamot polyphenol supplementation, recommended to subjects with metabolic syndrome, improve the lipid profile and fasting plasma glucose and reduce the HOMA-IR factor. Additionally, reductions in BW and BMI were observed in the high-dose supplementation group. Following these changes, a reduction in atherosclerotic risk was observed due to bergamot intake. What is important to say is that the changes were dose-dependent [130]. A lowering effect when it comes to uric acid levels was observed for a nutraceutical composed of bergamot and *Cynara cardunculus*, which is linked to a decrease in cardiovascular risk. This change was higher in subjects with moderate or severe liver steatosis [131]. A study conducted by Ferro et al. [132] on patients with liver steatosis revealed that bergamot in combination with *Cynara cardunculus* is effective in reducing hepatic fat; however, following weight change adjustment, this change was significant only in subjects over 50 years [132].

##### Cornelian Cherry

Cornelian cherry (*Cornux mas.* L.) belongs to the Cornaceae family and can be found in Europe and Asia. Its health benefits have been reported in studies, proving that it is effective in reducing HbA1c levels in diabetic patients, improving lipid profiles, and can be a factor that helps with weight loss. Among its constituents, anthocyanins, iridoids, phenolic acids, tannins, and flavonoids can be specified [133,134,135]. Studies conducted on NAFLD subjects have shown that cornelian cherry supplementation may possibly improve NAFLD patient states [134,135].

#### 3.2.2. Vegetables

##### Garlic

Garlic (*Allium sativum* L.) is a herb known in many cultures around the world. It is used in traditional medicine and is known to have positive beneficial effects as it is insulin sensitizing, lowers blood pressure, and improves lipid profiles. It has even been observed that garlic has anti-inflammatory properties, promotes weight loss, and can decrease hepatic fat content [136]. Among the garlic components, allicin, alliin, S-allyl mercaptocysteine, and S-allyl cysteine can be distinguished. Fermented garlic extracts are known as a factors that decrease the hepatic enzymes GGT and ALT, contributing to their beneficial impact on the state of the liver [137]. A study conducted on NAFLD patients has confirmed the role of garlic powder in decreasing liver enzyme levels and improving lipid profiles. In addition, a decrease in hepatic fat was observed due to garlic supplementation, which indicates it as being a potential plant-derived medication for dealing with NAFLD [138].

##### Purslane

Purslane (*Portulaca oleracea*) is a plant that grows in a lot of parts of the globe, including tropical and temperate areas. In TCM, it is used to treat diabetes, urolithiasis, and atherosclerosis; in Iranian traditional medicine, it is used to treat uterine bleeding. The useful parts of this plant are the seeds and leaves. Purslane contains flavonoids, vitamins E and C, omega-3 fatty acids, PUFA, and glycosides [139,140]. In addition, its seeds are rich in sitosterol, and, for this part of the plant, it was revealed that it has beneficial effects when it comes to lipid profiles, liver enzyme levels, and diabetes control in T2DM patients. Its efficiency is comparable to that of metformin [140]. Another study revealed improvements in HbA1c levels and blood pressure in T2DM patients as a result of purslane herb extract treatment [141]. In obese adolescents, a decrease in LDL and TG was observed due to purslane seed powder supplementation [139]. It is not surprising that researchers decided to take a closer look at purslane activity in NAFLD. Purslane seed intake in conjunction with a low-calorie diet in this group of patients led to a decrease in TC, LDL, QUICKI, and fasting plasma glucose levels [142]. In regard to purslane hydroalcoholic extract treatment in NAFLD, researchers observed enhancements in lipid enzyme levels, glycemic indices, TG, and LDL [143].

##### Artichoke

Artichoke (*Cynara Scolymus*) is a member of the daisy family. It is mainly found in the Mediterranean area, but it can also be found in other countries. It is used in Europe to treat digestive or urinary diseases, but its hepatoprotective action has also been observed. Its ingredients include flavonoids, bitters, and caffeoylquinic acids [144,145]. Researchers observed a decrease in plasma total cholesterol in patients supplemented with leaf extracts from this plant. However, LDL, HDL, and TG were not statistically changed in this study [145]. When it comes to NAFLD, Majnooni et al. [146] indicated the beneficial effects of artichoke leaf extract supplementation with metformin and vitamin E co-treatment on the NAFLD state. This co-treatment was more effective than only metformin or vitamin E when it came to decreasing TG and BMI and increasing HDL serum levels. In addition, a higher beneficial impact on reducing hepatic fat amounts was observed in subjects following artichoke supplementation with metformin and vitamin E than in the group co-treated with metformin and vitamin E [146]. There is also a study that checked the effects of artichoke leaf extracts without any addition on NAFLD subjects. At the endpoint of the intervention, NAFLD severity decreased, and improvements in the lipid profile, liver ultrasound parameters, and levels of hepatic enzymes in plasma were observed [147].

#### 3.2.3. Flowers and Spices

##### Sour Tea

Sour tea (*Hibiscus sabdariffa*) is an herbal medicine growing in the Middle East for which antitumor, anti-inflammatory, antioxidant, and antihypertensive properties have been observed. Among its constituents, alkaloids, anthocyanin, citric acid, galactose, flavonoids, polysaccharides, wax, -sitosterol, and -carotene have been identified. In traditional medicine, sour tea is used to treat fever, hepatic diseases, and hypertension. Studies performed on diabetic patients have revealed that sour tea supplementation has a beneficial impact on lipid profiles and blood pressure values [148,149,150]. A study conducted on NAFLD patients revealed beneficial outcomes in this group [151].

##### Magnolia

*Magnolia officinalis* is a tree, and certain parts, including the flowers or bark, derived from this plant and are used in traditional medicine in China and Japan to treat anxiety, gastrointestinal disorders, and allergic diseases. Within its components, honokiol, magnolol, and obovatol can be found [152,153]. It was revealed that honokiol has an anti-inflammatory action [154]. A study conducted on NAFLD patients revealed that the administration of an extract of *Magnolia officinalis* at a dose of 400 mg per day is an efficient option for reducing liver fat amounts. What is important to say is that a lower dose of this extract did not reveal such an effect on the mentioned parameter [155].

##### Cinnamon

Cinnamon is derived from the inner bark of the *Cinnamomum* tree, which can be found in South America, Asia, and Australia. It is known in TCM and has been used to treat diabetes mellitus. Cinnamon is rich in polyphenols, and its antioxidant, antitumoral, and antimicrobial properties are known [156,157]. A study conducted on diabetic patients revealed that adding cinnamon to gliclazide treatment results in a better metabolic control of diabetes, measured by a decrease in fasting plasma glucose and HbA1c. Even TG levels were reduced due to this intervention [157]. When it comes to lipid profiles, another meta-analysis is partially in line with these results and has revealed that cinnamon supplementation decreases the levels of TG and TC, but has no effect on LDL or HDL [156]. A study conducted on NAFLD patients confirmed cinnamon supplementation’s beneficial effects on lipid profiles and glucose maintenance. More improvements in liver enzymes and hs-CRP were shown. In sum, cinnamon may be a good additional therapy for this disease [158].

##### Cardamom

Cardamom is a spice obtained from *Elettaria cardamomum* fruit which belongs to the ginger family. Among its constituents, gallic acid, caffeic acid, quercetin, limonene, kaempferol, luteolin, and pelargonidin have been identified. Studies have revealed that cardamom extracts have anti-inflammatory, anti-proliferative, antimicrobial, anti-oxidative, and even anti-cancer properties. Due to these, it is not surprising that beneficial effects on infections, gastrointestinal diseases, and lung congestion have been observed [159,160,161]. A study conducted on T2DM patients showed that green cardamom supplementation may be an effective agent in improving metabolic parameters, including decreasing TG [160]. As positive effects of green cardamon on human health have been observed, researchers conducted a study on NAFLD subjects, which had a beneficial outcome in this group of patients. What should be emphasized is the fact that a decrease in fatty liver grade was observed due to this intervention [162].

##### Curcumin

Curcumin is a polyphenol found in a spice herb named turmeric (*Curcuma longa*), which is also used in medicine. Its hepatoprotective, anti-diabetic, anti-inflammatory, anti-oxidant, antimicrobial, and lipid profile properties are known [163,164]. In a group of overweight subjects, phytosomal curcumin supplementation resulted in improvements in anthropometric measures, HDL, TG, cortisol levels, insulin resistance, and fatty liver indices [164].

Multiple studies have revealed its usefulness in NAFLD-affected patients. Among its beneficial effects in this group, improvements in metabolomic profile, glycemic indices, lipid profile, indices of fatty liver grade, and hepatic enzyme levels have been specified [163,165]. On the other hand, data on its efficacy in improving HDL level and glycemia control parameters are not in line with another study, which did not reveal such changes [166]. It is worthy emphasizing that curcumin may be an effective factor leading to an improvement in liver fat content, which was revealed by researchers [165,167]. Saadati et al. [168] compared curcumin supplementation combined with lifestyle changes to the modification of lifestyle alone. Even if there is evidence of curcumin reducing liver fat amounts, this study did not prove its higher effectiveness in this parameter over lifestyle modification. However, it was revealed that curcumin combined with lifestyle modification is an effective factor when it comes to reducing liver fibrosis [168]. The anti-inflammatory properties of curcumin are known; however, data from studies are not clear. Saadati et al.’s [168] study did not prove that curcumin supplementation with lifestyle modification is more effective in reducing TNF-α levels than lifestyle changes alone [168]. On the other hand, Saberi-Karimian et al. [167] observed decreases in TNF-α, MCP-1, and EGF due to curcuminoid supplementation and indicated that these inflammatory parameter changes may have been related to the beneficial effects of curcuminoids on the liver’s state [167].

The studies on fruits, vegetables, flowers, and spices that have been conducted in NAFLD patients are presented in Table 2.

### 3.3. Other

#### 3.3.1. Citrulline

Citrulline is an amino acid for which it was revealed that it improves lipid metabolism in the liver. Rat models have revealed that citrulline supplementation reduces liver TG amounts and improves the lipid profile. Additionally, researchers have observed decreases in endoplasmic reticulum stress, the proinflammatory state, and insulin levels after citrulline supplementation. In addition, citrulline may be a preventive factor for NAFLD development [11,169,170]. One study conducted on NAFLD patients is in line with the mentioned rodent models, as decreases in inflammatory markers and liver fat amounts were observed after citrulline supplementation [171].

#### 3.3.2. Yogurts

Yogurt is a fermented dairy product. It contains a lot of vitamins, minerals, proteins, and phenolic compounds. Furthermore, lactose-intolerant patients generally tolerate yogurt well. In the fermentation process, lactic acid bacteria such as *Lactobacillus*, *Streptococcus*, *Pediococcus*, and *Leuconostoc* are the main types involved [172]. Yogurt is also known to have a beneficial effect on intestinal microbiota, for which connections with NAFLD development have been observed. The positive impact of yogurt intake has been confirmed by a lot of studies, and it is related to reducing obesity, metabolic syndrome, and diabetes risk. Its positive effects also apply to patients with NAFLD. A study conducted on NAFLD-diagnosed females revealed that yogurt is more efficient than milk when it comes to improving insulin resistance, the level of ALT, inflammatory parameters, and even reducing intrahepatic fat [173]. Yogurt can also be enriched with probiotics or symbiotics, which can improve yogurt’s effectiveness in terms of its effects on human health. In one study, researchers observed a decrease in NAFLD grade, measured with USG, due to symbiotic yogurt consumption, and it was significant compared to the non-symbiotic yogurt group. Additionally, improvements in liver enzymes were observed [174]. When it comes to hepatic enzyme levels, another study’s results are in line with those mentioned before, and due to probiotic yogurt intake, such outcomes were observed. Additionally, improvements in TC and LDL were shown after probiotic yogurt follow-up [175].

#### 3.3.3. Soy milk

Soybean (*Glycine max*) is a seed crop that was domesticated about 5000 years ago in China. Now it is known worldwide, and its consumption is rising [176]. Among its constituents, fiber, proteins, isoflavones, genistein, glycitein, daidzein, and unsaturated fatty acids have been identified [177]. It has been observed that soy protein intake has a positive impact on bone metabolism and may even affect body composition [178]. Studies conducted on patients suffering from NAFLD have revealed that soy milk may be a beneficial food for this group. Researchers have observed improvements in inflammatory state, glucose maintenance, blood pressure, and ALT levels in patients on calorie-restricted diets combined with soy milk use. These results indicate that even if weight loss is considered as the main treatment strategy for NAFLD, additional soy milk supplementation can increase the benefits gained from calorie restriction [177,179].

#### 3.3.4. Propolis

Propolis is a resinous substance produced by honeybees (*Apies mellifera*) from plant exudates, bud, wax, pollen, and bee enzymes. It is used by bees in their combs as a building and protection material due to its antimicrobial and antiseptic properties. Propolis is known to have antitumor, immunomodulatory, wound healing, and anti-inflammatory actions, which were revealed by lot of studies [180]. Among its constituents’ flavonoids, terpenoids, phenolics, sugar, hydrocarbons, and minerals can also be found. When it comes to the composition of propolis, it may vary depending on the bee species and geographical area [181]. Propolis was known about even in ancient times, and was used, e.g., by Egyptians to embalm their dead. Its properties mean it is used to deal with, for example, colds, wounds, diabetes, or rheumatism [180,181]. Recently, it was observed that propolis can be a good supplementary therapy for NAFLD due to its preventive effects on liver fibrosis and steatosis in NAFLD-diagnosed subjects [182].

#### 3.3.5. Omega Fatty Acids

The importance of omega-3 fatty acids intake has been revealed by a lot of studies, including those on NAFLD subjects and liver condition [183,184,185,186]. Omega-3 fatty acids can be found in many products, such as cereal products, fish, fish oils, lamb, seeds, and vegetable oils [187]. Among omega-3 fatty acids, eicosapentaenoic acid (EPA) and docosahexaenoic acid (DHA) have been identified. In one study, it was observed that 13–15 months of supplementation with Omacor (composed of 380 mg of DHA and 460 mg of EPA per 1 g of Omacor) in a dose of 4 g daily can reduce cardiovascular risk by decreasing prothrombin and apolipoprotein B-100 levels. In addition, effects on cholesterol metabolism, fasting TG concentrations, blood coagulation, and immune function were demonstrated. Moreover, hepatic lipid levels decreased as a result of DHA and EPA supplementation [184,185]. Other studies are in line with the results previously mentioned when it comes to improving liver fat content due to omega-3 intake alone, or synergistically alongside the decrease achieved by weight loss [188,189,190,191]. Song et al.’s [192] study revealed that better effects than DHA and EPA alone can be achieved by co-supplementing them with phytosterol ester, when it comes to an increase in the liver/spleen attenuation ratio, which proves that hepatic steatosis treatment is more effective. This study also revealed that EPA and DHA, even without phytosterol ester, decrease TNF-α and transforming growth factor beta (TGF-β). In addition, co-supplementation with EPA, DHA, and phytosterol ester is effective in improving TC and TG levels [192]. Within the beneficial effects of omega-3 supplementation in NAFLD subjects, improvements in visceral adiposity index, HDL, and decreases in LDL, TG, GGT, and ALT can be detailed [185,189,190,191]. A great source of EPA and DHA is fish oil. A study examining the effect of fish oil supplementation on the health of patients with NAFLD showed an improvement in metabolic parameters in this group of patients [193].

Other omega-3 fatty acids with beneficial health outcomes are α-linolenic acid (ALA) and linoleic acid (LA), which can also be found in fish, oils, or seeds [187]. A study conducted on NAFLD subjects revealed that conjugated LA co-administration with vitamin E (3000 mg + 400 IU, respectively, per day) and a weight loss diet result in improvements when it comes to HbA1c, the TC to HDL ratio, LDL to HDL ratio, and ALT to AST ratio. What is more, researchers have observed that this intervention has a positive impact on body fat and muscle mass [194]. When it comes to ALA, it was revealed that it is an effective factor in increasing adiponectin and decreasing IL-6 levels. These results are for co-supplementation with vitamin E at a dose of 400 IU per day with ALA at a dose of 1200 mg per day [195].

As we can see, omega-3 fatty acid supplementation may be a good path for the therapy of patients with NAFLD due to its broad effect on the metabolic profile, and reduction in the degree of fatty liver, which has been shown in numerous studies.

#### 3.3.6. Vitamins

The role of vitamins in human organisms is well known. They are responsible for antioxidant activity, metabolism regulation, wound healing, and immune system maintenance, among other things. They can be derived from food, for example, vegetables or milk, or they can be supplemented. Vitamin deficiencies are associated with consequences for the health of the body, but it should also be noted that in excess, vitamins can also be harmful [196]. Studies aiming to determine the usefulness of vitamins in NAFLD subjects have revealed positive outcomes due to the administration of vitamin D, B12, and E [197,198,199,200,201].

When it comes to vitamin D, it was revealed that due to supplementation with this substance, a decrease in liver steatosis, measured by CAP in subjects with this vitamin deficiency, was seen, even if liver function parameters and anthropometric measures were unchanged [200]. A study conducted on NAFLD overweight women revealed, in a 6-week follow-up to 2000 IU vitamin D per day supplementation, that vitamin D leads to improvements in weight, BMI, WHR, and body fat percentage. As the study was combined with eccentric exhaustive exercise training before and after vitamin D intervention, better outcomes in lipid profile, ALT, AST, and GGT were shown before and after the training at the end of the study compared to placebo [201]. In another study conducted on NAFLD patients, 4 months of supplementation with vitamin D at a dose of 50,000 IU each for 14 days resulted in a decrease in malondialdehyde, which is a marker of lipid peroxidation, and elevated levels of malondialdehyde are related to NAFLD [202]. On the other hand, another study on NAFLD subjects did not reveal the beneficial outcomes of cholecalciferol in NAFLD patients with type 2 diabetes when it came to changes in hepatic fat fraction, fatty liver index, and transaminases [203].

To our knowledge, there is only one study on 40 NAFLD subjects that aimed to determine vitamin B12 effectiveness. After 12 weeks of 1000 μg cyanocobalamin supplementation, a significant decrease in homocysteine was observed compared to placebo. Researchers also showed declines in ALT, FBG, MDA, and liver steatosis, but these changes did not reach significance when compared to placebo [197]. Due to the scarcity of data on the effects of vitamin B12 on NAFLD, more studies are needed in larger populations to determine its usefulness.

We mentioned the usefulness of vitamin E in co-supplementation with other substances in previous paragraphs. Vitamin E is also known as α-tocopherol, and δ-tocotrienol, in turn, is a naturally occurring subtype of this vitamin. It was revealed that vitamin E supplementation leads to a decline in ALT in subjects with NAFLD [199]. A study aiming to compare the effectiveness of δ-tocotrienol and α-tocopherol on NAFLD subjects in doses of 300 mg and 268 mg, respectively, twice a day revealed improvements in fatty liver index, liver-to-spleen ratio, HOMA-IR, and malondialdehyde following 48-week supplementation. In addition, δ-tocotrienol seemed to be more effective when it came to increasing in adiponectin and decreasing IL-6, TNF- α, weight, leptin, and cytokeratin-18 [204]. These data are in line with another study comparing δ-tocotrienol with placebo, which showed improvements in liver steatosis and biochemical markers of hepatocellular damage after this treatment [205].

The studies on citrulline, yogurts, soy milk, propolis, omega-3 fatty acids, and vitamins that have been conducted in NAFLD patients are presented in Table 3.

#### 3.3.7. Food-Based Compositions

As we have described in previous paragraphs, the impact of given substances on the health status of patients with NAFLD was undoubtedly the starting point for the creation of functional foods based on some of these products. A lot of studies confirmed the usefulness of using functional foods in the context of NAFLD treatment. Among beneficial outcomes, improvements in anthropometric measures, liver function, hepatic fat amounts, and glucose maintenance parameters can be specified [206,207,208]. To provide better clarity, we will present the composition of the given nutraceuticals and their impact on the condition of patients with NAFLD in the form of a table.

D-002, composed of high-molecular-weight beeswax alcohols, appeared effective in reducing liver steatosis, insulin sensitivity, and enhancing antioxidant properties in NAFLD patients. Patients felt better NAFLD syndrome relief with D-002 when compared to placebo. Triacontanol, which is present in D-002 and activates adenosine monophosphate (AMP)-kinase, may be associated with insulin desensitization. Even though patients were advised to follow a weight-loss diet, no weight loss was observed at the end of the study, indicating that the reported results are more likely the result of D-002 as opposed to diet [206].

In a study conducted by Ferro et al. [207], another supplement, Livogen Plus^®^, appeared effective in reducing liver obesity in NAFLD patients, particularly in men aged 60 or younger. Moreover, subjects with reduced HDL levels at the beginning of the study demonstrated greater improvement. This study was also conducted on cultured liver cells, which revealed that Livogen’s polyphenols reduce the lipid fat content of hepatocytes. However, the authors indicate that this nutraceutical only produces positive results in subjects with severe NAFLD, indicating that it should only be used in this group, and that its effectiveness in preventing NAFLD is rather limited [207].

The study by Abidov et al. [208] on the nutraceutical XanthigenTM revealed its anti-obesity properties in obese women with NAFLD. As a factor that contributes to reductions in body fat and liver fat, it may play an essential role in the prevention and treatment of NAFLD. Importantly, this role in NAFLD may be associated with weight loss induced by this supplement or be directly attributable to a decrease in hepatic fat levels. The authors indicate that its anti-inflammatory effect may be facilitated by weight loss. In addition, XanthigenTM increased resting energy expenditure, which may be an important driver leading to weight loss [208].

The authors who evaluated the efficacy of Eufortyn^®^ Colesterolo Plus in healthy individuals with polygenic hypercholesterolemia found improvements in lipid profile, fatty liver index, endothelial dysfunction, and systemic inflammation at the endpoint. This nutraceutical appears to be a potential preventative agent for NAFLD development [209].

In NAFLD patients, Viusid supplementation with diet and exercise has been shown to ameliorate the activity score of NAFLD, liver steatosis, lobular inflammation, and hepatocyte ballooning in NAFLD patients. In comparison to the placebo group, the outcomes were more pronounced, indicating that Viusid is an excellent additional therapy that may enhance the benefits of lifestyle modification in NAFLD [210].

The studies on nutraceuticals that have been conducted in NAFLD patients are presented in Table 4.

## 4. Probiotics, Prebiotics, and Synbiotics

Recent studies have focused on the significance of the gut–liver axis in the pathogenesis of NAFLD. The systemic and portal circulatory systems, as well as the biliary duct, connect these two organs. The intestinal microbiome is a vital component of human health. It has been observed that an unhealthy diet high in fat and low in fiber degrades the microflora condition and may contribute to the development of liver diseases [211]. Changes in gut permeability allow intestinal microorganisms, their metabolomics, or inflammatory markers to access the liver. It is not remarkable that it has recently become a focal point for NAFLD treatment, as it has been shown that intestinal microbiota improvement may have positive effects on liver health [9].

### 4.1. Probiotics and Prebiotics

Probiotics are characterized as live microorganisms that provide a health benefit to the host when administered in adequate amounts [212]. Symbiter, a combination of probiotic species from 14 different genera, was found to be effective in improving NAFLD symptoms. A lower FLI was achieved at the endpoint in comparison to the placebo group. In addition, there was evidence of a reduction in liver enzymes and TG. A decrease in proinflammatory cytokines also appeared to have affected the inflammation [213]. Taking a probiotic supplement including *Lactobacillus casei*, *Lactobacillus rhamnosus*, *Lactobacillus acidophilus*, *Bifidobacterium longum*, and *Bifidobacterium breve* daily in conjunction with a lifestyle modification can reduce TG levels and liver enzymes after 12 weeks of treatment [214]. Probiotic supplementation may lead to reductions in intrahepatic fat amounts and TG levels in obese subjects with NAFLD, according to another study. However, even if the changes in these parameters were significant, they were no longer significant after adjusting for weight loss, highlighting the importance of weight loss in the treatment of NAFLD. Nevertheless, probiotic supplementation may enhance the results of weight loss [215]. In contrast, a follow-up study on people with NAFLD who were given a probiotic blend combining *Lactobacillus* and *Bifidobacterium* species (MCP^®^ BCMC^®^ strains) found no improvement in liver steatosis, fibrosis, or the lipid profile. Nevertheless, the scientists suggest that this probiotic can help to cure NAFLD patients by reducing gut permeability issues [8].

A prebiotic is a substrate that confers health benefits to the host microorganisms as it is used by them selectively. Among these advantages is a better functioning digestive system as a result of immune system stimulation and pathogen suppression. However, it is also known that it has positive effects on cardiometabolism, or mental and bone health [216]. An examination in NAFLD patients of whether the prebiotic inulin, taken after a short course of metronidazole, can help people keep off the weight they have lost on a diet found no significant benefits over placebo. However, the prebiotic group showed further ALT reduction [217]. In another study, liver enzyme and lipid profile improvements were observed after 12 weeks of oligofructose supplementation [214].

### 4.2. Synbiotics

The effects of single-handed probiotic or prebiotic supplementation in NAFLD have been examined and positive outcomes of such techniques have been demonstrated. However, another method of supplementation is a synbiotic combination of the above-mentioned preparations. A 12-week follow-up to supplementation with *Bacillus coagulans* and inulin resulted in a decrease in liver steatosis, which was followed by improvements in several liver enzymes and inflammatory markers in the NAFLD group [218]. The quoted data are consistent with another synbiotic investigation. Although the amount of liver fat was not measured, a drop in liver fibrosis score was found after 28 weeks of taking Protexin capsules daily. Improvements in liver enzymes and inflammatory indicators were detected in a manner similar to the previously mentioned results [219]. Even though the majority of NAFLD patients are overweight, it also impacts lean individuals. A subsequent study assessed the effect of synbiotics on this patient population. Liver fibrosis and steatosis improved after supplementation with Protexin and a healthful lifestyle for 28 weeks. This study suggests that the improvement in NAFLD through the use of synbiotics may be associated with an inflammatory process pathway [220].

In one word, there are mounting indications that probiotics, prebiotics, and synbiotics are beneficial in the treatment of NAFLD. On the basis of cited data, it appears that synbiotics may be the most beneficial, particularly Protexin, which reduced liver fibrosis and steatosis in slender NAFLD patients. However, these studies were conducted on relatively small populations, but they indicate relationships that can be used in future research. The studies of probiotics, prebiotics, and synbiotics that have been conducted on NAFLD patients are presented in Table 5.

## 5. Limitations of Studies Focused on Food-Derived Treatment Strategies for NAFLD

The previously cited and analyzed studies shed new light on NAFLD treatment. Nonetheless, it is important to note that there are some limitations associated with them, which can impact the final outcome of a patient’s condition. At first glance, it appears that these studies were conducted using relatively limited sample sizes, with only a few involving more than 100 patients. A larger scale of research will reduce the likelihood of random outcomes and provide stronger evidence of the substance’s utility. Additionally, the length of specific studies may indicate the need to extend them. A more extensive analysis over time will permit a more in-depth evaluation of long-term effects or the danger of side effects or drug interactions. What is equally important is that it will allow for the standardization of the doses of the substances used. Often, research on a given substance is carried out together with other interventions, such as changing the diet, reducing body weight, or increasing physical activity. This, in our opinion, complicates the final analysis, as the results obtained at the conclusion of the study may be attributable to other interventions besides supplementation. In conclusion, we believe that diet-based therapies have the potential to become an integral part of NAFLD treatment. There is evidence of their utility, but additional large-scale and long-term research is required to obtain firmer proof. As a result, the strength of clinical evidence will increase, and it will be easier to justify theories about the usefulness of a given method. The aforementioned information can serve as a starting point or a suggestion for future researchers interested in the topic of NAFLD therapy using functional foods.

## 6. Conclusions

**Figure 2:**
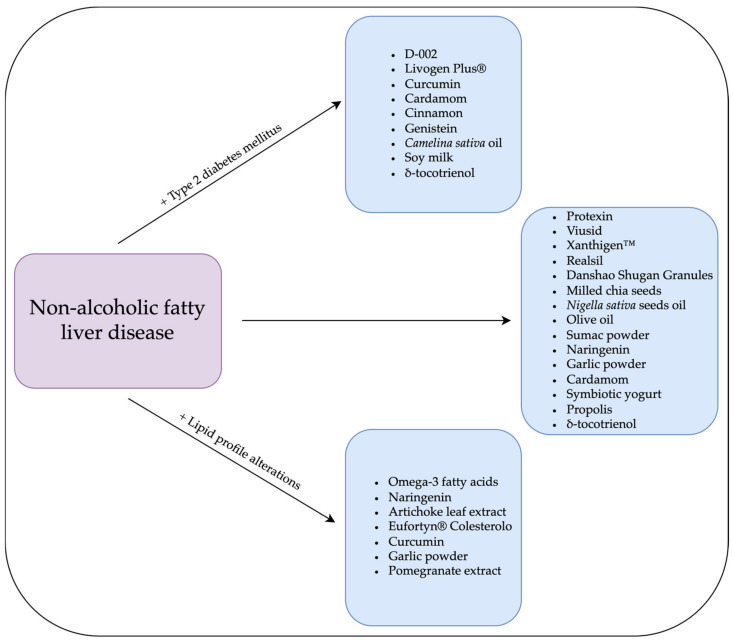
Proposed supplementary treatment strategy for NAFLD.

The global prevalence of NAFLD and the complications it brings within require a standardization of treatment recommendations. This paper is aimed at defining the usefulness of food-derived substances in NAFLD supplementary therapy, which can be an addition to lifestyle modifications. Undoubtedly, maintaining an optimal body weight is crucial for staying in good health and preventing the development of disorders such as NAFLD. It can also slow the progression of NAFLD. It is important for the beneficial outcomes resulting from weight loss to be increased using additional strategies such as supplementation. Noteworthy is the combination of a hypocaloric diet with sesame or olive oil intake, due to its benefits in terms of fatty liver grade. Among the substances we have discussed, those used in ancient times can provide new achievements in the world of science in the form of nutraceuticals. Some of them lead to a reduction in the amount of fat in the liver, some have a better effect on anthropometric parameters, and others affect the lipid profile. Therefore, the choice of supplementation therapy should be tailored to the patient’s needs and overall health. This work collectively and transparently presents the effects of given substances on parameters in patients with NAFLD. These food-derived supplements are readily available and can be used even by people at risk of developing NAFLD to prevent its occurrence. A physician’s knowledge of supplements of natural origin will allow them to advise patients, for example, on what products to introduce to their diet as a supplement to other treatment methods to increase its effectiveness. Nevertheless, despite many studies on supplements in NAFLD therapy, it seems crucial to conduct research on a larger scale, over a longer period of time, and in a larger group of subjects. As an additional treatment for NAFLD, it appears that numerous food-derived substances are effective. However, in our opinion, Protexin, D-002, Viusid, and omega-3 fatty acids deserve a distinction in terms of the treatment of NAFLD. It is important to note that due to the wide range of activities of different substances, we must adapt their use to the patient’s requirements, as some substances reduce inflammatory parameters more effectively than others, while others are more effective in patients with concomitant diabetes. A proposed food-derived additional treatment strategy for NAFLD patients is presented in Figure 2.

## Figures and Tables

**Table 1:** The efficiency of plant supplementation in NAFLD patients.

Study	Group *	NAFLD Diagnosis	Substance	Duration	Results **	Possible Mechanism
Loguercio et al. [29] (2012)	138 patients with NAFLD (69 in treatment group)	Histology–liver biopsy	Realsil (94 mg of silybin and 194 mg of phosphatidylcholine) + 89.28 mg vitamin E acetate 50% (30 mg of α-tocopherol)	12 months	Normalization of ALT and AST ↓: GGT, HOMA-IR, improvement in liver histology	Antioxidant activity which may lead to decline in membrane lipid peroxidation and restoration of glutathione levels
Wang et al. [31] (2022)	260 patients with NAFLD (130 in the DSSG group)	B-ultrasound	DSSG in 12 g package, 2 packages/time, 3 times per day	16 weeks	↓: TC, TG, ALT, AST, GGT, FPG Improvement in B-USG	Inhibition of lipid peroxidation; decline in NF-κB expression in liver
Chen et al. [37] (2015)	60 patients with NAFLD (30 in treatment group)	B-ultrasound	Dihydromyricetin in dose 150 mg twice a day	3 months	↑: Adiponectin ↓: ALT, AST, GGT, LDL-C, apo-B, HOMA-IR, TNF- α, CK-18, FGF-21	Prevention of hepatocyte apoptosis; regulation of antioxidant properties; decline in proinflammatory cytokine levels
Zhang et al. [40] (2015)	74 patients with NAFLD (1:1 with placebo)	USG	Anthocyanin in dose 80 mg in capsule, two capsules twice a day	12 weeks	↓: ALT, CK-18, myeloperoxidase, 2 h OGTT, plasma glucose and HOMA-IR	Antioxidant and anti-inflammatory properties; improvement in IR
Medina-Urrutia et al. [43] (2020)	25 patients with NAFLD	CT imaging	Milled chia seeds 25 g per day	2 weeks of dietary stabilization and 8 weeks of chia supplementation	↑: L: SAR, ALA plasma concertation, dietary fiber consumption ↓: VAF, BW, BMI, WC, TC, non-HDL, FFA	Fiber may lead to improvements in intestinal barrier which may further promote intestinal transit slowdown, production of GLP-1, and favor the sensation of satiety
Zamani et al. [48] (2018)	85 patients with NAFLD (45 in treatment group)	USG	Zataria multiflora powder 700 mg twice a day	12 weeks	↓: Serum insulin, IR, SBP, DBP	IR, and serum TG levels improvements through increase in PPAR-γ expression; increase in adiponectin leads to gluconeogenesis decline
Khonche et al. [58] (2019)	120 patients (60 patients in treatment group)	USG	2.5 mL standardized *Nigella sativa* seed oil every 12 h	3 months	↑: HDL-C ↓: Grade of hepatic steatosis, ALT, AST, TG, LDL-C	PPAR-γ upregulation; anti-inflammatory, antifibrotic and antioxidant properties
Musazadeh et al. [64] (2021)	43 patients with NAFLD (22 in CSO group)	USG	*Camelina sativa* oil in dose 20 g per day + calorie restricted diet	12 weeks	↑: HDL-C ↓: hs-CRP, insulin, HOMA-IR, QUICKI, LPS, TAC, SOD, GSH-Px, MDA, 8-iso-PGF2α	Omega-3 fatty acids in CSO may lead to induction of GLP-1; CSO leads to decrease in energy intake, and inflammation
Arefhosseini et al. [69] (2022)	40 overweight/obese females with NAFLD (21 in HCA group)	USG	*Garcinia cambogia* extract (HCA) in connection with calorie restriction diet	8 weeks	↑: HDL-C ↓: BW, WC, HC, FPG, LDL-C, TG, TG/HDL-c ratio	Improvement in lipid profile due to suppression of ATP-citrate lyase and appetite; enhancement of glycogen deposition in the liver; anti-obesity properties due to regulation of serotonin levels, reduction in de novo lipogenesis, reduction in leptin, and insulin levels in plasma
Ebrahimi-Mameghani [71] et al. (2014)	55 NAFLD patients (29 in intervention group)	USG	*Chlorella vulgaris* 300 mg in tables per day + vitamin E 400 mg per day	8 weeks	↓: BW, liver enzymes, FPG, ALP, TG, TC, LDL-C	Possible effect related to BW reduction; improvements in glucose metabolism by boost of glucose uptake
Cossiga et al. [76] (2019)	49 NAFLD patients (26 in plant extracts supplementation group)	Transient elastography	*Berberis aristata* (588 mg), *Elaeis guineensis* (143 mg) and decaffeinated green coffee by *Coffea canephora* (67 mg) extracts in dose 1 table per day	6 months	↓: serum glucose, insulin, HOMA-IR, CAP	Modulation of serum insulin receptor levels by activation of protein kinase C; insulin sensitizing effect through the activation of AMPK
Sakata et al. [81] (2013)	17 patients with NAFLD (12 in green tea supplementation group)	USG and CT	Green tea in dose 700 mL per day, containing above 1 g catechin	12 weeks	↑: L: SAR ↓: BF, ALT, urinary 8-isoprostane excretion	Decline in hepatic oxidation stress; inhibition of lipase which leads to decrease in glucose and fat absorption; improvements in liver lipid metabolism by increase in mRNA expression of peroxisomal, and mitochondrial β-oxidizing enzymes
Amanat et al. [86] (2018)	78 patients with NAFLD (41 in genistein group)	USG	Genistein in dose 250 mg per day	8 weeks	↓: Serum insulin, HOMA-IR, MDA, TNF-α, IL-6, WHR, BF, TG	Increase in glucose uptake by promotion of glucose transporter type 4 translocation to a membrane; activation of AMPK; upregulation of genes related to antioxidant properties by NF-κB and Nrf2 transcription factors
Atefi et al. [90] (2022)	53 females with NAFLD (27 in sesame oil group)	USG	Sesame oil supplementation in dose 30 mg per day + low-calorie diet	12 weeks	↓: BW, BMI, WC, fatty liver grade, AST, ALT	Inhibiting matrix metalloproteinases-2, 9 activities and upregulating PPAR-γ expression; antioxidant properties; reduction in lipogenic enzymes mRNA expression, and induction of mRNA expression of enzymes related to fatty acid oxidation, e.g., CoA dehydrogenases, acyl-CoA oxidase, or 3-hydroxyacyl CoA dehydrogenase
Rezaei et al. [94] (2019)	54 patients with NAFLD (26 in olive oil group)	USG	Olive oil in dose 20 g per day + recommendation of hypocaloric diet	12 weeks	↓: Fatty liver grade in USG, BW, WC, BP, AST, TG, fat mass	Antioxidant properties; action of omega-3 fatty acids; increase in post-prandial β-oxidation of fatty acids; improvements in IR
Schweinlin et al. [101] (2018)	36 patients with NAFLD (17 in oat enriched diet group)	FLI	Restricted diet + oat intake	12 weeks	↓: BMI, hepatorenal index, BP	Improvements in gut microbiota, which may be related to fiber intake
Lee et al. [105] (2019)	76 patients with NAFLD (27 in pinitol high dose group)	USG	Pinitol in dose 500 mg per day	12 weeks	↑: GSH-Px ↓: Liver fat amount, MDA, AST, ALT, GGT, postprandial TG	Increase in liver antioxidant enzyme activities, e.g., GSH; decline in the rate of glutathione turnover
Kazemi et al. [107] (2020)	80 patients with NAFLD (1:1 with placebo)	Hepatic fibrosis grade based on FibroScan device and ALT level	Sumac powder in dose 2 g per day + calorie deficit diet	12 weeks	↑: QUICKI ↓: liver fibrosis score, ALT, AST, hs-CRP, MDA, FPG, HbA1c, HOMA-IR, serum insulin	Inhibition of α-glucosidase and pancreatic α-amylase; suppression of lipogenic factor genes; stimulation of AMPK and lipolysis; increase in PPAR-γ gene expression
Yan et al. [110] (2015)	155 patients with NAFLD (55 in berberine group)	Proton magnetic resonance spectroscopy	Berberine in dose 500 mg 3 times per day + lifestyle intervention	16 weeks	↓: HFC, BW, WC, HOMA-IR, TC, TG, ALT	Mechanism is still unclear, but may be linked to expression of genes related to glucose and lipid metabolism such as CPT-1, GCK, or MTTP
Amerikanou et al. [114] (2021)	98 patients with NAFLD (41 in mastiha group)	LiverMultiScan technique	Mastiha in dose 350 mg three times per day	6 months	Positive effects on intestinal microbiota, improvement in lipid metabolite levels Additionally, in severely obese subjects, ↓ liver fibrosis score, liver inflammation	Reduction in expression of genes related to collagen (Col1a1 and Col4a1); decline in inflammatory and endotoxin-producing bacteria; increase in anti-inflammatory bacteria

Abbreviations: 8-iso-PGF2α–8-iso-prostaglandin F2α, ALA—alpha linolenic acid, ALP—alkaline phosphatase, ALT—alanine aminotransferase, AMPK—5′-adenosine monophosphate-activated protein kinase, apo-B—apolipoprotein B, AST—aspartate aminotransferase, BF—body fat, BMI—body mass index, BP—blood pressure, BW—bodyweight, CAP—controlled attenuation parameter, CK-18—cytokeratin-18, CT—computed tomography, DBP—diastolic blood pressure, DSSG—Danshao Shugan Granules, FFA—free fatty acids, FGF21—fibroblast growth factor 21, FPG—fasting plasma glucose, GGT—γ-glutamyltranspeptidase, GLP-1—glucagon-like peptide-1, GSH-Px—glutathione peroxidase, HbA1c—glycated hemoglobin, HC—hip circumference, HCA—hydroxycitric acid, HDL-C—high density lipoprotein cholesterol, HFC—hepatic fat content, HOMA-IR—homeostasis model assessment of insulin resistance, hs-CRP—high sensitive C-reactive protein, IL-6—interleukin 6, LDL-C—low density lipoprotein cholesterol, L:SAR—liver to spleen attenuation ratio, IR—insulin resistance, LPS—lipopolysaccharide, MDA—malondialdehyde, NAFLD—non-alcoholic fatty liver disease, Nrf2—Nuclear factor erythroid 2-related factor 2, OGTT—oral glucose tolerance test, PPARγ—peroxisome proliferator activated receptor protein, QUICKI—quantitative insulin sensitivity check index, SBP—systolic blood pressure, SOD—Superoxide dismutase, TAC—total antioxidant capacity, TC—total cholesterol, TG—triglycerides, TNF-α—tumor necrosis factor alpha, USG—ultrasonography, VAF—visceral abdominal fat, WC—waist circumference, WHR—waist to hip ratio. * In the group column, the number of respondents analyzed was given, not the people who were included in the study and for various reasons were excluded from it during procedures. ** Changes shown in results column are significant.

**Table 2:** The efficiency of fruit, vegetable, flower, and spice supplementation in NAFLD patients.

Study	Group *	Diagnosis	Substance	Duration	Results **	Possible Mechanism
Hormoznejad et al. [121] (2020)	41 patients with NAFLD (20 in cranberry group)	USG	Vaccinium macrocarpon extract in tablets (144 mg) twice a day + weight-loss diet	12 weeks	↓: ALT, insulin, HOMA-IR	Decrease in hepatic inflammation, as evidenced by decreased TNF- α, NF-κB, COX2, and IκB mRNA expression; inhibition of release of TNF- α, IL-1β, IL-6, and IL-8 from lipopolysaccharide
Goodarzi et al. [124] (2021)	44 patients with NAFLD (22 in pomegranate extract group)	USG	Pomegranate extract in dose 225 mg twice a day	12 weeks	↑: HDL-C ↓: TC, TG, LDL to HDL ratio, FPG, HOMA-IR, DBP, BMI, BW, WC	Anti-obesity effect gained by decrease in pancreatic lipase activity and energy intake, reduction in fat absorption and elevation of its fecal secretion; agonist action on PPAR-α and PPAR-γ; decrease in resistin secretion
Namkhah et al. [127] (2021)	44 patients with NAFLD (22 in naringenin group)	USG	Naringenin in dose 100 mg twice a day	4 weeks	↑: HDL-C ↓: BW, BMI, TG, TC, LDL-C, NAFLD grade	Inhibition of overproduction of VLD; modification of expression of genes related to lipid metabolism: upregulation the expression of PPAR-α and PPAR-γ genes, modulation of CPT1α, SREBF1c genes, and HMG-CoA reductase; alterations in collagen deposition, modulation of oxidative stress and inflammatory process
Ferro et al. [132] (2020)	86 patients with liver steatosis (45 in intervention group)	Transient elastography	Bergamot polyphenol fraction and Cynara Cardunculus extract in dose 300 mg per day	12 weeks	↓: BW, BMI CAP score decreased only in subjects over 50 years	Regulating the potential of the mitochondrial membrane and oxidative phosphorylation; upregulation of antioxidant-related genes; anti-inflammatory properties
Sangouni et al. [138] (2020)	88 patients with NAFLD (45 in garlic group)	USG	400 mg of garlic powder four times a day	12 weeks	↑: HDL-C ↓: Liver steatosis, ALT, GGT, TC, TG, LDL	Lipogenesis modulation by lowering the activity of enzymes involved in hepatic fat synthesis; decrease in insulin resistance, activity of NF-κB pathway, and oxidative stress
Damavandi et al. [143] (2021)	71 patients with NAFLD (37 in purslane group)	USG	Purslane extract, 300 mg once a day	12 weeks	↓: ALT, AST, GGT, FPG, TG, LDL-C	Improvements in glucose uptake and fatty acid metabolism; increase in insulin secretion from β-cells; activation of AMPK and PI3K pathways in skeletal muscles; upregulation of GLUT-4 gene expression; enhancing GLP-1 concentration; inhibition of pancreatic lipase activity, decrease in ACC activity
Panahi et al. [147] (2018)	81 patients with NAFLD (41 in artichoke group)	USG	Artichoke leaf extracts in dose 600 mg per day	2 months	↑: Hepatic vein flow, AST/ALT ratio ↓: Portal vein diameter, liver size, ALT, AST, total bilirubin, LDL-C, TC, TG, HDL-C, non-HDL-C, BMI, WC, APRI	Enhancement of expression of the following genes: malic enzyme 1, decorin, cytochrome P450, family 1, subfamily a, polypeptide 2 and nicotinamide phosphoribosyltransferase, which leads to improvements in fatty acids metabolism, inflammation, and liver fibrosis
Izadi et al. [151] (2021)	61 patients with NAFLD (30 in sour tea group)	USG	Sour tea powder in capsule in dose 450 mg	8 weeks	↓: TG, ALT, AST, SBP, DBP, TAC	Mechanisms are not fully understood; probably improvements are due to an inhibition of fatty acid synthesis, decrease in lipogenesis, improvements in mitochondrial activity and β-oxidation, decrease in production of reactive oxygen species, proinflammatory cytokines and chemokines in liver
Jeong et al. [155] (2017)	60 patients with NAFLD (20 in 400 mg dose of HL)	USG	Magnolia officinalis extract–HL tablet in dose 400 mg per day	12 weeks	↓: HFC	Anti-inflammatory and antioxidant properties; induction of apoptosis of the activated hepatic stellate cells; inhibition of liver toxicity and lipid accumulation
Askari et al. [158] (2014)	45 patients with NAFLD (23 in intervention group)	USG	Cinnamon in dose 750 mg twice a day	12 weeks	↓: HOMA-IR, FPG, TC, TG, ALT, AST, GGT, hs-CRP	Reduction in post-prandial glucose absorption gained due to inhibition of pancreatic α-amylase and α-glucosidase; stimulation of glucose metabolism and glucose uptake by improving glucose transporter-4; decrease in gluconeogenesis and improvement in IR
Daneshi-Maskooni et al. (2019) [162]	87 patients with NAFLD (43 in cardamom group)	USG	Cardamom in dose 1 g three times per day with meals	3 months	↑: Irisin, HDL, QUICKI ↓: fasting insulin, TG, HOMA-IR, fatty liver grade	Increase in antioxidant capability, PPAR-γ expression; decrease in inflammation and cholesterol synthesis; improvement in IR
Rahmani et al. [165] (2016)	77 patients with NAFLD (37 in curcumin group)	USG	Amorphous dispersion curcumin formulation in dose 500 mg per day (equal to 70 mg of curcumin)	8 weeks	↓: HFC, BMI, TC, LDL-C, TG, ALT, AST, FPG, HbA1c	Inhibition of oxidative stress and NF-κB; downregulation of pro-inflammatory cytokines, COX-2, collagen 1, inducible nitric oxide synthase, and ICAM-1; improvements in acyl-CoA oxidase activity and lipid peroxidation through glutathione level modulation

Abbreviations: ACC—acetyl-coA carboxylase, ALT—alanine aminotransferase, AMPK—5′-adenosine monophosphate-activated protein kinase, APRI—aspartate aminotransferase to platelet ratio index, AST—aspartate aminotransferase, BMI—body mass index, BW—bodyweight, CAP—controlled attenuation parameter, COX2—cyclooxygenase-2, CPT1α—carnitine palmitoyltransferase 1α, DBP—diastolic blood pressure, FPG—fasting plasma glucose, GGT—γ-glutamyltranspeptidase, GLP-1—glucagon-like peptide-1, GLUT-4—glucose transporter-4, HbA1c—glycated hemoglobin, HDL-C—high density lipoprotein cholesterol, HFC—hepatic fat content, HMG-CoA—3-hydroxy-3-methylglutaryl-Coenzyme A, HOMA-IR—homeostasis model assessment of insulin resistance, hs-CRP—high sensitive C-reactive protein, ICAM-1—intracellular adhesion molecule-1, IL—interleukin, LDL-C—low density lipoprotein cholesterol, NAFLD—non-alcoholic fatty liver disease, NF-κB—nuclear factor kappa-light-chain-enhancer of activated B cells, PI3K—phosphoinositide 3-kinase, PPAR—peroxisome proliferator activated receptor protein, QUICKI—quantitative insulin sensitivity check index, SBP—systolic blood pressure, TAC—total antioxidant capacity, TC—total cholesterol, TG—triglycerides, TNF-α—tumor necrosis factor alpha USG—ultrasonography, VLDL—very low-density lipoprotein, SREBF1c—sterol regulatory element-binding transcription factor-1, WC—waist circumference. * In the group column, the number of respondents analyzed is given, not the people who were included in the study and for various reasons were excluded from it during procedures. ** Changes shown in results column are significant.

**Table 3:** The efficiency of citrulline, yogurts, soy milk, propolis, omega-3 fatty acids, and vitamin supplementation in NAFLD patients.

Study	Group *	Diagnosis	Substance	Duration	Results **	Possible Mechanism
Darabi et al. [171] (2019)	44 patients with NAFLD (22 in citrulline group)	Fibroscan	Citrulline in dose 500 mg four times a day	12 weeks	↓: TNF-α, hs-CRP, NF-κB, ALT, liver steatosis	Reduction in TLR4 gene expression may lead to decrease in NF-kB activation and TNF-α production; decrease in oxidative stress by increase in SOD may lead to reduction in ERK1/2 signaling activation, and what follows this is a decrease in NF-kB activity and TNF-α production; protection against hepatic lipid peroxidation
Bakhshimoghaddam et al. [174] (2018)	102 patients with NAFLD (34 in symbiotic yogurt group)	USG	300 g of symbiotic yogurt (containing 108 colony-forming units Bifidobacterium animalis/mL and 1.5 g inulin) per day	24 weeks	↓: NAFLD grade (USG), ALT, AST, ALP, GGT	Modulation of intestinal microbiota, and improvements in gut barrier; improvements in insulin resistance; improvements in post-prandial absorption of micro- and macronutrients, including antioxidants; increased production of GLP-2
Maleki et al. [177] (2019)	62 patients with NAFLD (31 in soy milk group)	USG	Soy milk in dose 240 mL per day + 500 calorie-deficit diet	8 weeks	↑: QUICKI ↓: Serum insulin, SBP, DBP, HOMA-IR	Decrease in intestinal alpha-glucosidase and protein tyrosine kinase activity; increased glucose uptake by glucose transporter type 4; inhibition of lipogenesis by decrease in ChREBP, SREBP1, LXR, and RXR expression
Soleimani et al. [182] (2021)	54 patients with NAFLD (27 in propolis group)	Elastography	Propolis tablets (250 mg twice a day)	4 months	↓: Liver stiffness, hs-CRP	Impact on cholesterol metabolism by upregulation of Apo A1 and ATP-binding cassette transporters A1 and G1 gene expression; inhibition of HMG-COA reductase gene expression; anti-inflammatory properties due to Nrf-2 activation, inhibition of IκBα, JNK, ERK1/2, and p38MAPK phosphorylation
Qin et al. [193] (2015)	70 patients with NAFLD (36 patients in fish oil group)	USG	Fish oil in 1g capsules, two capsules twice per day	3 months	↑: Adiponectin ↓: TG, TC, apolipoprotein B, glucose, ALT, GGT, TNF- α, FGF21, leukotrienes B, cytokeratin 18 fragment M30, prostaglandin E2	Anti-inflammatory effects, decrease in NF-kB activity; inhibition of prostaglandin E2 leads to improvements in lipid profile
Rahimpour et al. [201] (2022)	22 overweight women with NAFLD (11 in vitamin D group)	USG	Vitamin D in dose 2000 IU per day + eccentric exhaustive exercise training at the beginning and at the endpoint	6 weeks	↓: BW, BMI, WHR, body fat percentage	Increase in lipoprotein lipase activity; inhibition of serum parathyroid hormone; improvement in calcium homeostasis as calcium affects bile acids to excrete fatty acids; improvement in IR; inhibition of lipogenesis and improvement in fatty acid oxidation
Pervez et al. [205] (2020)	71 patients with NAFLD (35 in δ-tocotrienol group)	USG	δ-tocotrienol in dose 300 mg twice a day	24 weeks	↓: FLI, HOMA-IR, hs-CRP, MDA, ALT, AST, hepatic steatosis	Upregulation of PPAR α and δ, and downregulation of fatty acid biosynthesis enzymes, e.g., HMGC, which leads to inhibition of adipogenesis; decrease in leptin level; increase in adiponectin

Abbreviations: ALP—alkaline phosphatase, ALT—alanine aminotransferase, AST—aspartate aminotransferase, ATP—Adenosine triphosphate, BMI—body mass index, BW—bodyweight, ChREBP—carbohydrate responsive element binding protein, DBP—diastolic blood pressure, ERK 1/2—extracellular signal-regulated protein kinases 1 and 2, FGF21—fibroblast growth factor 21, FLI—fatty liver index, GGT—γ-glutamyltranspeptidase, GLP—glucagon-like peptide, HMGCR—3-hydroxy-3-methylglutaryl-CoA reductase, HOMA-IR—homeostasis model assessment of insulin resistance, hs-CRP—high sensitive C-reactive protein, IκBα—nuclear factor of kappa light polypeptide gene enhancer in B-cells inhibitor alpha, JNK—c-Jun N-terminal kinase, LXR—liver X receptor, MDA—malondialdehyde, NAFLD—non-alcoholic fatty liver disease, NF-κB—nuclear factor kappa B, Nrf2—nuclear factor erythroid 2-related factor 2, QUICKI—quantitative insulin sensitivity check index, p38MAPK—p38 mitogen-activated protein kinase, PPAR—peroxisome proliferator-activated receptor, RXR—retinoid-X-receptor, SBP—systolic blood pressure, SOD—superoxide dismutase, SREBP1—sterol-regulatory element binding protein-1, TC—total cholesterol, TG—triglycerides, TGF-β—transforming growth factor-beta, TLR4—Toll-like receptor 4, TNF- α—tumor necrosis factor alpha, USG—ultrasonography, WHR—waist to hip ratio. * In the group column, the number of respondents analyzed was given, not the people who were included in the study and for various reasons were excluded from it during procedures. ** Changes shown in results column are significant.

**Table 4:** The efficiency of nutraceutical supplementation in NAFLD patients.

Study	Nutraceutical	Composition	Group *	Diagnosis	Duration	Results **	Possible Mechanism
Illnait et al. [206] (2013)	D-002–substance derived from beeswax in dose 50 mg twice a day + low fat, low energy diet	Mixture of six of the higher aliphatic alcohols (C26, C26, C28, C30, C32, and C34)	44 patients with NAFLD (22 in D-002 group)	USG	24 weeks	↑: Plasma total antioxidant status ↓: HOMA-IR, insulin, liver steatosis Normalization of liver echo pattern in USG (7 patients), symptoms improvement (12 patients)	Antioxidant properties; improvement of IR probably through AMP-kinase activation, which leads to metabolic gene regulation and decline of AMP-mediated insulin secretion by pancreatic β-cells
Ferro et al. [207] (2022)	Livogen Plus^®^ 6 capsules per day	Doses for 6 capsules: 667 mg of Curcuma longa extract complexed with γ-cyclodextrin, 667 mg of refined fish oil concentrate, 400 mg of BPF and wild type Cynara Cardunculus extract, 334 mg of black seed oil of *Nigella sativa*, 267 mg of standardized fraction of root of Picrorhiza kurroa, 200 mg reduced GHS and SAMe, 167 mg of Artichoke leaf extract, Indole-3-carbinol and silybin phospholipids, 127 mg of milk thistle fruit dry extract, and other natural compounds	127 patients with NAFLD (62 in Livogen Plus group)	Transient elastography	12 weeks	↓: CAP, DBP, insulin, HOMA-IR Improvement in liver steatosis shown only in subjects with severe NAFLD	Antioxidant properties; prevention of lipid peroxidation; stimulation of mitochondrial β-oxidation
Abidov et al. [208] (2010)	Xanthigen™ 3 capsules per day + calorie restricted diet	100 mg pomegranate seed oil + 100 mg brown marine algae containing 0.8 mg fucoxanthin	72 obese females with NAFLD (36 in Xanthigen group)	Magnetic hepatic ultrasound scanning + image-guided proton magnetic resonance spectroscopy	16 weeks	↓: BW, WC, body fat content, HFC, CRP, TG, ALT, AST, GGT	Promotion of hepatic fatty acids β-oxidation; increase in resting energy expenditure; weight loss, which promotes decrease in inflammatory cytokines
Fogacci et al. [209] (2022)	Eufortyn^®^ Colesterolo Plus 1 tablet per day + 1-month Mediterranean diet standardization before start of the study	Doses per tablet: 1000 mg of Phytosome Bergamot Polyphenolic fraction, 100 mg of Cynara cardunculus L. dry extract, 20 mg of Cynara scolymus L. dry extract, 25 mg of Coenzyme Q10 phytosome and 5 mg of zinc	56 healthy subjects with polygenic hypercholesterolemia (28 in nutraceutical group)		8 weeks	↓: TC, LDL-C, LDL-C to HDL-C ratio, hs-CRP, WC, lipid accumulation products, FLI	Anti-inflammatory and antioxidant properties; inhibition of HMG-CoA reductase; interaction with liver SREPBs and ACAT; increase in bile acids excretion to feces; improvements in endothelial function; regulation of VEGF, ET-1, eNOS genes expression
Vilar Gomez et al. [210] (2009)	Viusid oral sachets 50 g per day + hypocaloric diet + aerobic exercise	666 mg of malic acid 33 mg of glycyrrhizic acid 666 mg of glucosamine 2 mg of calcium pantothenate 20 mg of ascorbic acid 666 mg of arginine 333 mg of glycine 66 μg of folic acid 600 mg of pyrodoxal 0.3 μg of cyanocobalamine 5 mg of zinc sulphate	60 patients with NAFLD (30 in Viusid group)	Liver biopsy + histology	6 months	↓: NAS, histological: steatosis, hepatocyte ballooning and lobular inflammation	Viusid mechanisms of action remain unclear however may be related to improvements in methylation reactions in hepatocytes, e.g., production of glutathione and improvements in cell membrane integrity, anti-inflammatory, and antioxidant properties

Abbreviations: ACAT—acetyl-CoA C-acetyltransferase, ALT—alanine aminotransferase, AMP—adenosine monophosphate, AST—aspartate aminotransferase, BPF—bergamot polyphenol fraction, BW—bodyweight, CAP—controlled attenuation parameter, CFU—colony-forming units, CRP—C-reactive protein, DBP—diastolic blood pressure, eNOS—endothelial NO synthase, ET-1—endothelin 1, FLI—fatty liver index, GGT—γ-glutamyltranspeptidase, GHS—glutathione, HDL-C—high density lipoprotein cholesterol, HFC—hepatic fat content, HMG-CoA—3-hydroxy-3-methylglutaryl-Coenzyme A, HOMA-IR—homeostasis model assessment of insulin resistance, LDL-C—low density lipoprotein cholesterol, NAFLD—non-alcoholic fatty liver disease, NAS—NAFLD activity score, VEGF—vascular endothelial growth factor, SAMe—S-adenosyl-l-methionine, SREBP—sterol regulatory element-binding protein, TC—total cholesterol, TG—triglycerides, USG—ultrasonography, WC—waist circumference. * In the group column, the number of respondents analyzed was given, not the people who were included in the study and for various reasons were excluded from it during procedures. ** Changes shown in results column are significant.

**Table 5:** The efficiency of probiotics, prebiotics, and synbiotics supplementation in NAFLD patients.

Study	Preparate + Dosage	Group *	Diagnosis	Duration	Results **	Possible Mechanism
Kobyliak et al. [213] (2018)	Symbiter (14 genera’s of probiotic species: *Bifidobacterium*, *Lactobacillus*, *Lactococcus*, and *Propionibacterium*) in dose 10 g per day	58 patients with T2DM and NAFLD (30 in probiotic group)	USG	8 weeks	↓: FLI, AST, GGT, TNF-α, IL-6, TG	Improvements in intestinal microbiota and gut permeability; decrease in JNK activity in liver; reduction in inflammatory markers
Ahn et al. [215] (2019)	Probiotic composed with: *Lactobacillus acidophilus*, *L. rhamnosus, L. paracasei*, *Pediococcus pentosaceus*, *Bifidobacterium lactis*, and *B. breve*	68 obese NAFLD patients (32 in probiotics group)	MRI	12 weeks	↓: IHF, TG However, after adjusting to BW changes, there were no differences between probiotic and placebo groups	Correction of intestinal dysbiosis by production of lactic acids, promotion of molecular immunity, and changes in intestinal environment
Abhari et al. [218] (2020)	10^9^ *Bacillus coagulans* spore (GBI-30) plus 0.4 g inulin per day	53 patients with NAFLD (22 in symbiotic group)	Fibroscan	12 weeks	↓: liver steatosis, ALT, GGT, TNF-α, NF-κB	Amelioration of inflammation by reducing pathogenic bacteria in gut and improving intestinal barrier
Eslamparast et al. [219] (2014)	Protexin containing 7 strains of bacteria: (*Lactobacillus casei*, *Lactobacillus rhamnosus*, *Streptococcus thermophilus*, *Bifidobacterium breve*, *Lactobacillus acidophilus*, *Bifidobacterium longum*, and *Lactobacillus* bulgaricus), fructooligosaccharide, probiotic cultures magnesium stearate and a vegetable capsule	58 patients with NAFLD (26 in symbiotic group)	Fibroscan	28 weeks	↓: liver fibrosis score, TNF-α, NF-κB, hs-CRP, GGT, ALT, AST,	Modulation of intestinal microbiota; reduction in serum inflammatory cytokines levels; decrease in JNK and NF-κB activity; improvement in IR through decline of endotoxins and intestinal toxins levels and increasing pH of feces
Mofidi et al. [220] (2017)	Protexin (the composition same as line above)	50 lean NAFLD patients (25 in symbiotic group)	Fibroscan	28 weeks	↓: liver fibrosis, liver steatosis, FPG, AST hs-CRP, NF-κB	

Abbreviations: ALP—alkaline phosphatase, ALT—alanine aminotransferase, AST—aspartate aminotransferase, BW—body weight, FLI—fatty liver index, FPG—fasting plasma glucose, GGT—γ-glutamyltranspeptidase, hs-CRP—high sensitive C-reactive protein IHF—intrahepatic fat, IL-6—interleukin 6, JNK—c-Jun N-terminal kinase, LDL-C—low density lipoprotein cholesterol, NF-κB—nuclear factor- κB, T2DM—type 2 diabetes mellitus, TG—triglycerides, TNF-α—tumor necrosis factor alpha, USG—ultrasonography. * In the group column, the number of respondents analyzed was given, not the people who were included in the study and for various reasons were excluded from it during procedures. ** Changes shown in results column are significant.

## Data Availability

Not applicable.

## Supplementary Materials

The following supporting information can be downloaded at https://www.mdpi.com/article/10.3390/nu15132838/s1, Figure S1: PRISMA diagram of the screening process in the systematic literature review; Table S1: Prisma checklist for systematic review.
